# Combination treatment with a PI3K/Akt/mTOR pathway inhibitor overcomes resistance to anti-HER2 therapy in *PIK3CA*-mutant HER2-positive breast cancer cells

**DOI:** 10.1038/s41598-020-78646-y

**Published:** 2020-12-10

**Authors:** Yumi Fujimoto, Tomoko Yamamori Morita, Akihiro Ohashi, Hiroshi Haeno, Yumi Hakozaki, Masanori Fujii, Yukie Kashima, Susumu S. Kobayashi, Toru Mukohara

**Affiliations:** 1 Department of Breast and Medical Oncology, National Cancer Center Hospital East, Kashiwa, Japan; 2 Division of Translational Genomics, Exploratory Oncology Research and Clinical Trial Center, National Cancer Center, Kashiwa, Japan; 3 Department of Computational Biology and Medical Sciences, Graduate School of Frontier Sciences, The University of Tokyo, Kashiwa, Japan; 4 Department of Medicine, Beth Israel Deaconess Medical Center/Harvard Medical School, Boston, MA USA

**Keywords:** Targeted therapies, Breast cancer

## Abstract

Amplification and/or overexpression of human epidermal growth factor receptor 2 (HER2) are observed in 15–20% of breast cancers (HER2+ breast cancers), and anti-HER2 therapies have significantly improved prognosis of patients with HER2+ breast cancer. One resistance mechanism to anti-HER2 therapies is constitutive activation of the phosphoinositide 3-kinase (PI3K) pathway. Combination therapy with small-molecule inhibitors of AKT and HER2 was conducted in HER2+ breast cancer cell lines with or without *PIK3CA* mutations, which lead to constitutive activation of the PI3K pathway. *PIK3CA* mutations played important roles in resistance to single-agent anti-HER2 therapy in breast cancer cell lines. Combination therapy of a HER2 inhibitor and an AKT inhibitor, as well as other PI3K pathway inhibitors, could overcome the therapeutic limitations associated with single-agent anti-HER2 treatment in *PIK3CA*-mutant HER2+ breast cancer cell lines. Furthermore, expression of phosphorylated 4E-binding protein 1 (p4EBP1) following the treatment correlated with the antiproliferative activities of the combination, suggesting that p4EBP1 may have potential as a prognostic and/or efficacy-linking biomarkers for these combination therapies in patients with HER2+ breast cancer. These findings highlight potential clinical strategies using combination therapy to overcome the limitations associated with single-agent anti-HER2 therapies in patients with HER2+ breast cancer.

## Introduction

Gene amplification, overexpression, and mutation of human epidermal growth factor receptor 2 (HER2), a transmembrane tyrosine kinase, are observed in approximately 20% of breast cancers and are associated with poor prognosis^1,2^. Oncogenic activation of HER2, driven by these gene alternations, constitutively activates downstream signaling pathways, such as the phosphoinositide 3-kinase (PI3K)/AKT/mammalian target of rapamycin (mTOR) pathway (also called the PI3K pathway), which is involved in metabolism, growth, survival, and protein synthesis^3^, and the RAS/RAF/mitogen-activated ERK kinase (MEK)/extracellular signal-regulated kinase (ERK) pathway (also called the mitogen-activated protein kinase [MAPK] pathway), which is involved in gene expression, mitosis, differentiation, proliferation, and cell survival/apoptosis^4^. During the last two decades, development of anti-HER2 therapies has significantly improved the prognosis of patients with HER2-positive (HER2+) breast cancer both at early and metastatic disease stages^5^; however, some patient populations demonstrate clinical resistance to anti-HER2 therapies^6^. One mechanism of resistance to anti-HER2 therapies is the constitutive activation of the PI3K pathway, which can occur through mutations/amplification of the *PI3K* p110α subunit (*PIK3CA*), loss of *PTEN*, and mutations/amplification of *AKT*^7,8^. Among these oncogenic alternations, *PIK3CA* mutations are observed in approximately 20–30% of patients with breast cancer, and cause resistance to anti-HER2 therapies in both preclinical and clinical settings^9–16^.

Several chemical inhibitors targeting the PI3K pathway have been developed as candidate anticancer therapies^17^. Evidence from preclinical and clinical studies suggests that combination treatment with anti-HER2 therapies and PI3K pathway inhibitors may have potential efficacy in HER2+ breast cancers with *PIK3CA* mutations^18–20^. However, clinical proof of concept for these combination therapies requires confirmation in further studies. Of these novel agents, AKT inhibitors have attracted attention as next-generation PI3K pathway inhibitors. Ipatasertib is an ATP-competitive small molecule pan-AKT inhibitor (AKT1, AKT2, and AKT3)^21^, and clinical proof of concept has been confirmed in a phase II clinical trial in which ipatasertib significantly improved progression-free survival (PFS) compared with placebo when combined with paclitaxel in patients with advanced triple negative breast cancers with *PIK3CA* mutation or PTEN loss^22^. Taken together, these data suggest that AKT inhibitors may have clinical potential in combination with anti-HER2 therapy, and that this combination may overcome the limitations associated with anti-HER2 therapy in patients with HER2+ breast cancer carrying *PIK3CA* mutations, and a highly-activated PI3K pathway.

In this preclinical study, we investigated combination therapy with small-molecule inhibitors of AKT and HER2 to overcome limitations associated with anti-HER2 monotherapy in HER2+ breast cancer cell lines with *PIK3CA* mutations. We also demonstrated that expression of phosphorylated 4E-binding protein 1 (p4EBP1), a downstream target of the PI3K pathway, may have potential as an efficacy-linking marker of combination treatment with AKT and HER2 inhibitors in patients with HER2+ breast cancer with *PIK3CA* mutations. These preclinical findings support the therapeutic potential of combination treatment with an AKT inhibitor and HER2 therapies in patients with HER2+ breast cancer carrying *PIK3CA* mutations.

## Results

### Analysis of overall survival stratified by PI3K pathway status in patients with HER2+ breast cancer

**Figure 1:**
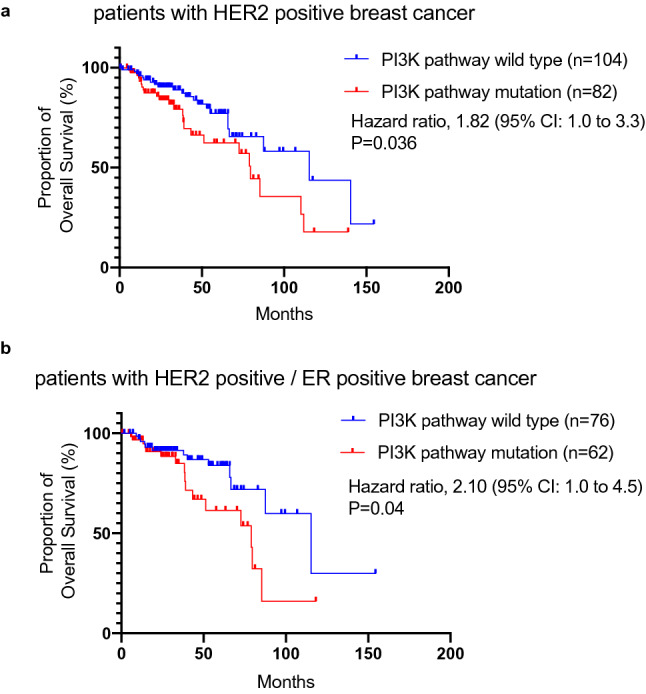
Mutations in the PI3K pathway are associated with poor overall survival for patients with HER2-positive (HER2+) and HER2 and ER double-positive (HER2+/ER+) breast cancer. (**a**) Overall survival (OS) was analyzed in patients with HER2+ breast cancer with (red) or without (blue) mutations in the PI3K pathway. Median OS was 115.3 (blue) vs 79.5 months (red), respectively (hazard ratio: 1.82; 95% CI 1.0–3.3, *p* value = 0.036). (**b**) OS was analyzed in patients with HER2+/ER+ breast cancer with (red) or without (blue) mutations in PI3K pathway. Median OS was 115.3 (blue) vs 79.0 months (red), respectively (hazard ratio: 2.10; 95% CI: 1.0–4.5, *p* value = 0.04).

Although several trials have reported that patients with PIK3CA mutant have poor prognosis as previously mentioned, some trials have reported that *PIK3CA* mutations were not significantly associated with resistance to anti-HER2 antibody therapies, such as TH3RESA trial treated with trastuzumab emtansin^23^, and NeoSphere trial with pertuzumab^12^. The clinical significance of resistance to anti-HER2 therapies associated with PI3K pathway activation remains unclear. To evaluate the clinical impact of a constitutively-activated PI3K pathway in patients with *PIK3CA, AKT, PIK3R1* mutation*,* and *PTEN* homozygous deletion or mutations, we retrospectively reanalyzed a large and unbiased clinical dataset of anti-HER2 therapies in the HER2+ metastatic or recurrent breast cancer patients^24^ (Supplementary Table S1). Of 186 HER2+ patients treated with anti-HER2 therapy, 44.1% possessed mutations of genes in the PI3K pathway; *PIK3CA* mutations were the most commonly observed (Table 1). Patients double-positive for HER2 and ER (HER2+ /ER+) also exhibited a similar frequency of PI3K pathway alterations (44.9%). The distribution of genomic alterations is shown in Oncoprint (Supplementary Fig. 1). Median OS in HER2+ patients with PI3K pathway alterations was significantly shorter than in those without PI3K pathway alterations (115.3 vs 79.5 months, respectively; hazard ratio, 1.82; 95% CI, 1.0–3.3; *p* = 0.036) (Fig. 1a). In the HER2+/ER+ patients, in addition, the PI3K pathway alternations significantly caused shorter mOS (115.3 vs 79.0 months, respectively; hazard ratio, 2.10; 95% CI, 1.0–4.5; *p* = 0.04) (Fig. 1b). These clinical observations suggest that oncogenic alterations in the PI3K pathway are associated with poor prognosis in patients with HER2+ breast cancer receiving HER2 therapy.

**Table 1:** Characteristics of the patient cohort selected from Razavi et al.^23^.

Characteristics	All patients (n = 186)	Estrogen receptor positive (n = 138)
Age at diagnosis*—yr	48 (40–55)	47 (39–54)
**PI3K pathway status—no.**		
Wild type	104 (55.9%)	76 (55.1%)
PIK3CA mutation	68 (36.6%)	51 (37.0%)
PIK3R1 mutation	6 (3.2%)**	3 (2.2%)
AKT1 mutation	1 (0.5%)***	1 (0.7%)***
PTEN homozygous deletion or mutation	9 (4.8%)	8 (5.8%)

*Shown as median (first and third quartiles).

**One patient has both PIK3CA and PIK3R1 mutations.

***One patient has both PIK3CA and AKT1 mutations.

### *PIK3CA* mutations attenuate the antiproliferative effects of a HER2 inhibitor and an AKT inhibitor enhances the antiproliferative activity of a HER2 inhibitor in *PIK3CA*-mut HER2+ breast cancer cell lines

To evaluate the effects of oncogenic activation in the PI3K pathway on anti-HER2 therapy, we performed in vitro proliferation assays using a HER2 inhibitor, lapatinib, in HER2+ breast cancer cell lines. Given that the PI3K pathway alterations have a high impact in patients with ER+/HER2+ breast cancer (Fig. 1), we first evaluated cell viability using ER+/HER2+ breast cancer cell lines with or without *PIK3CA* mutations. In this study, BT474 and MDA-MB-361 cell lines were used, which represent *PIK3CA*-wt and *PIK3CA*-mut (E545K) ER+/HER2+ breast cancer cell lines, respectively. BT474 cells possess a non-canonical *PIK3CA* mutation at K111N, which would preclinically and clinically contributes to less, or exceptionally-mild, activation of PI3K and its down-stream pathways^22,25^. (Supplementary Fig. 2). Due to the limited numbers of the commercially-available ER+/HER2+ breast cancer cell line, we used BT474 cells as a surrogate cell line model for PIK3CA-wt ER+/HER2+ breast cancer cell. Since a combination of ER and HER2 therapy is clinically-used for the treatment of patients with ER+/HER2+ breast cancer, we also applied an ER inhibitor fulvestrant and a HER2 inhibitor lapatinib to the preclinical studies of in vitro cell viability assays. Lapatinib was also selected as a HER2 inhibitor in the following studies, because the preclinical studies using lapatinib are well established due to its usability. In addition, although lapatinib is a dual EGFR/HER2 inhibitor, the EGFR signaling is expected to have little contribution to proliferative activities in HER2+ breast cancer cells^26,27^.

**Figure 2:**
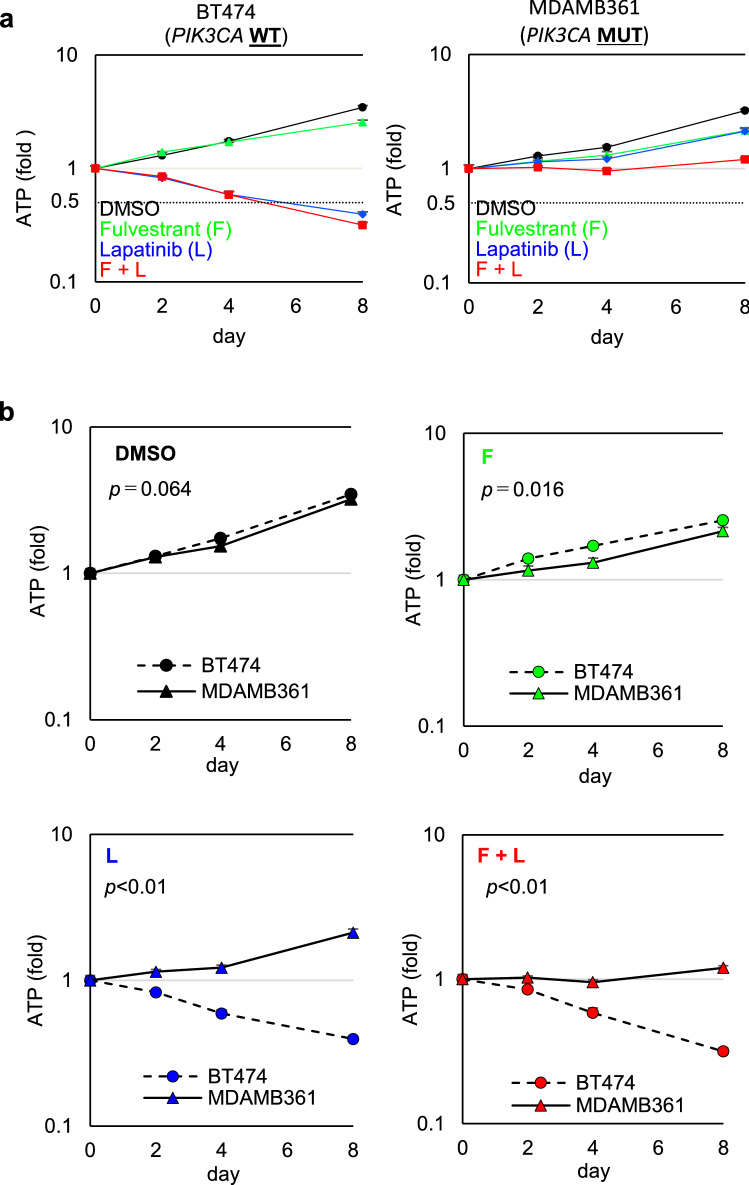
The antiproliferative effect of treatment with fulvestrant and lapatinib is limited in MDA-MB-361 cell line. (**a**) Antiproliferative activity of fulvestrant and lapatinib, alone and in combination, in ER+/HER2+ breast cancer cell lines with and without *PIK3CA* mutations. BT474 (left) and MDA-MB-361 (right) cells were used as representative PIK3CA-wild-type and PIK3CA-mutant ER+/HER2 breast cancer cell lines, respectively. Cells were treated with DMSO (black), fulvestrant (100 nM, green), or lapatinib (100 nM, blue), alone and in combination (red), for 0, 2, 4, and 8 days (mean ± standard deviation [SD; n = 3]). Relative levels of ATP were calculated by chemiluminescence assay and compared with the chemiluminescence of DMSO on day 0. (**b**) Comparison of the effect in each drug treatment between BT474 and MDA-MB-361 cell lines. Differences on day 8 were analyzed using Student’s t-test. The data represent the mean ± SD (n = 3). F, Fulvestrant; L, Lapatinib.

**Figure 3:**
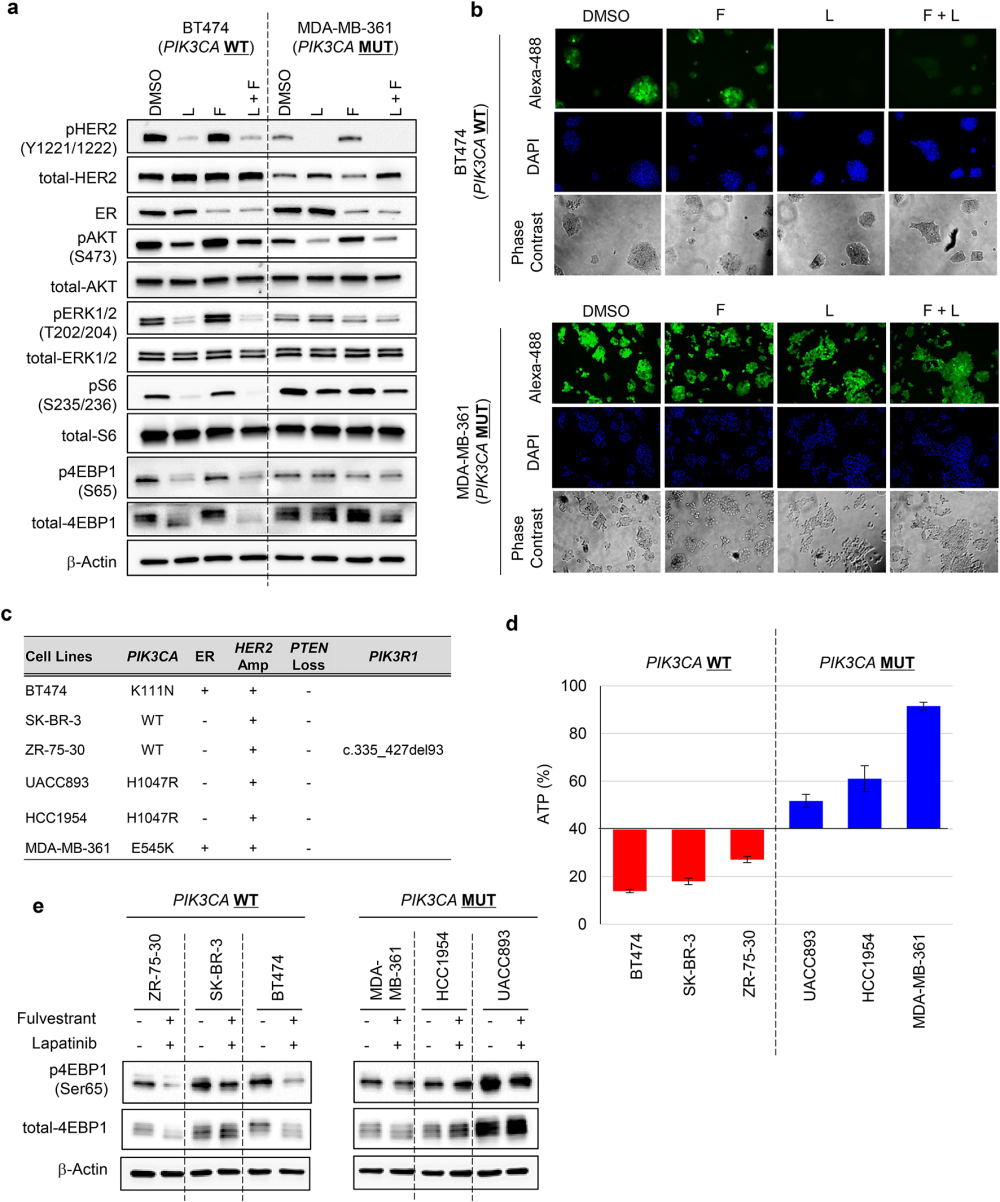
Lapatinib suppresses 4EBP1 phosphorylation insufficiently in MDA-MB-361 cells and the antiproliferative effect of lapatinib is limited in HER2+ breast cancer cell lines with *PIK3CA* mutations. (**a**) Inhibitory effects of fulvestrant (100 nM) and lapatinib (100 nM), alone and in combination treated for 24 h, on 4EBP1 phosphorylation in ER+/HER2+ breast cancer cell lines with and without *PIK3CA* mutation. Full-length blots are presented in Supplementary Fig. 3. (**b**) Newly synthesized protein in DMSO-, fulvestrant-, lapatinib-, and combination-treated cells. BT474 (upper) and MDA-MB-361 (lower) cells were treated with the indicated inhibitors for 24 h; Click-iT HPG was incorporated into the drug-treated cells for 15 h. Green indicates the newly synthesized proteins detected by the Click-iT assay (Life Technologies) and DAPI-stained nuclei, respectively. (**c**) *PIK3CA* mutation, ER expression, HER2 amplification, PTEN Loss and PIK3R1 status of the breast cancer cell lines used in this study. Information on gene modification and expression was obtained from the COSMIC database and ATCC information. (**d**) Antiproliferative activity of combined fulvestrant and lapatinib in HER2+ breast cancer cell lines with and without PIK3CA mutation. Three PIK3CA-wild-type (BT474, SK-BR-3, and ZR-75-30; red bars) and three PIK3CA-mutant (UACC893, HCC1954, and MDA-MB-361; blue bars) HER2+ breast cancer cell lines were used. The cells were treated with fulvestrant and lapatinib in combination for 8 days (mean ± SD [n = 3]). Y-axis indicates relative amounts of ATP (%), which were calculated with a chemiluminescence assay and compared with the chemiluminescence value of DMSO treatment on day 8. The data represent the mean ± SD (n = 3). (**e**) Inhibitory effects of fulvestrant (100 nM) and lapatinib (100 nM) in combination treatment for 24 h, on 4EBP1 phosphorylation in HER2+ breast cancer cell lines with and without *PIK3CA* mutation. Three PIK3CA-wild-type (BT474, SK-BR-3, and ZR-75-30) (left panel) and three PIK3CA-mutant (UACC893, HCC1954, and MDA-MB-361) (right panel) HER2+ breast cancer cell lines were used. Full-length blots are presented in Supplementary Fig. 4.

BT474 and MDA-MB361 cells were treated with fulvestrant alone, lapatinib alone, or a combination of both for 0, 2, 4, and 8 days. The viability of drug-treated cells was determined by assessing intercellular ATP levels (Fig. 2a). Protein expression of ER and phosphorylation of HER2 at Tyr1221/1222 (pHER2) were used to confirm target engagement for fulvestrant and lapatinib, respectively (Fig. 3a). Phosphorylated ERK (pERK) at Thr202/204 was used as a biomarker of signal modulation within the MAPK pathway. Phosphorylated 4EBP1 at Ser65 (p4EBP1) and phosphorylated S6 at Ser235/236 (pS6) were used as biomarkers of signal modulation within the PI3K pathway. Immunoblotting analysis confirmed that fulvestrant and lapatinib treatments potently inhibited their cellular targets in both BT474 and MDA-MB-361 cells (Fig. 3a). Consistent with previous reports^28,29^, pHER2 and/or its downstream targets (pERK or p4EBP1) were mildly upregulated in fulvestrant-treated cells (Fig. 3a).

The cell viability assay revealed that treatment with fulvestrant alone exerted on no antiproliferative effects in BT474 cells (Fig. 2a, left panel, green line,), while treatment with lapatinib alone or in combination of lapatinib and fulvestrant demonstrated potent antiproliferative effects in a time-dependent manner, leading to growth regression (Fig. 2a, left panel, blue and red lines,). In those cells, treatment with lapatinib alone or in combination with fulvestrant effectively inhibited pERK and p4EBP1, both of which are downstream targets of HER2. In contrast, lapatinib alone or in combination with fulvestrant showed limited antiproliferative effects and failed to induce tumor regression in MDA-MB-361 cells (Fig. 2a, right panel, blue and red lines). Comparison of the effect in each drug treatment between BT474 and MDA-MB-361 cell lines (Fig. 2b) also revealed that lapatinib treatment in either a single-agent (Fig. 2b, lower-left panel) or a fulvestrant-combination (Fig. 2b, lower-right panel) significantly induced a highly-potent anti-proliferative effects in BT474 cells, while the growth rates in DMSO treatment were equivalently (Fig. 2b, upper-left panel). Immunoblotting analysis revealed that, although pHER2 was completely inhibited, substantial amounts of p4EBP1 and pS6 were present in MDA-MB-361 cells treated with lapatinib alone or in combination with fulvestrant. In MDA-MB-361 cells, furthermore, expression level of pERK was relatively low and pERK modification was also less affected in treatment with lapatinib, implying that proliferation in MDA-MB-361 cells may be more addicted to the PI3K pathways than the ERK pathway. These results suggest that lapatinib failed to completely inhibit PI3K signaling, as the *PIK3CA* mutation leads to constitutive activation of the PI3K pathways in MDA-MB-361 cells, thus resulting in less inhibition, or only partial inhibition, of p4EBP1 and pS6.

Since p4EBP1 positively regulates protein synthesis though Eukaryotic translation initiation factor 4E (eIF4E)^7,21^, we next determined how newly-synthesized proteins are modulated by lapatinib to identify difference between BT474 and MDA-MB-361 cells. The results of the Click-iT assay revealed the presence of newly-synthesized proteins in MDA-MB-361 cells following treatment with lapatinib; conversely lapatinib alone or in combination with fulvestrant potently inhibited protein synthesis in BT474 cells (Fig. 3b, upper). These data suggest that inactivation of HER2 kinase by lapatinib fails to fully inhibit PI3K signaling, and partial activation of PI3K attenuates antiproliferative activity of lapatinib in *PIK3CA*-mut cells. Furthermore, to confirm that constitutive activation of PI3K signaling caused by *PIK3CA*-mutations attenuates the antiproliferative effects of HER2 inhibition regardless of ER status, we tested four additional ER−/HER2+ breast cancer cell lines: SK-BR-3 cells and ZR-75-30 cells (*PIK3CA*-wt/ER−/HER2+), and UACC893 cells and HCC1954 cells (both *PIK3CA*-mut [H1047R]/ER−/HER2+) (Fig. 3c). The results of a cell viability assay revealed that antiproliferative activity measured by ATP was more potent in *PIK3CA*-wt/HER2+ breast cancer cell lines (Fig. 3d, red bars) than in *PIK3CA*–mut HER2+ breast cancer cell lines (Fig. 3d, blue bars). Additionally, inhibitory effect of lapatinib on p4EBP1 was evaluated in these cell lines (Fig. 3e). In accordance with the results in BT-474 and MDA-MB-361 cells (Fig. 3a), lapatinib treatment also exhibited less inhibition of p4EBP1 in the *PIK3CA*–mut HER2+ cells compared to the *PIK3CA*–wild HER2+ cells (Fig. 3e). Taken together, these results demonstrate that the residual expression of p4EBP1 in the post-treatment, which is mediated by *PIK3CA* mutations, would attenuate the antiproliferative effects of lapatinib in both ER+/HER2+ and ER−/HER2+ breast cancer cell lines, supporting the clinical results demonstrating relatively poor prognosis in patients with HER2+ breast cancer patients with active mutations in the PI3K pathway regardless of the status of ER expression (Fig. 1).

**Figure 4:**
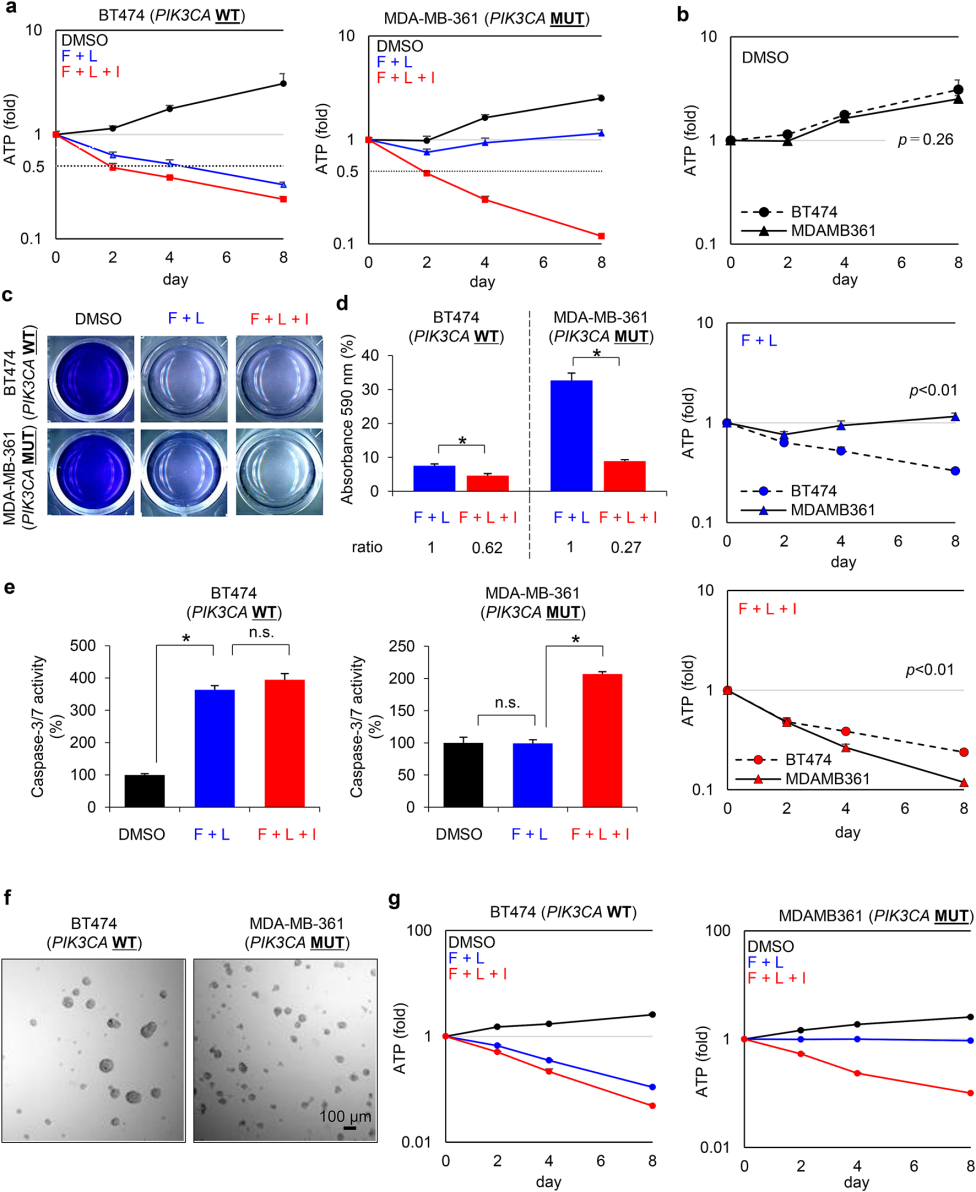
Ipatasertib enhances the antiproliferative activity of fulvestrant and lapatinib combination in a PIK3CA-mutant HER2+/ER+ breast cancer cell line. (**a**) Antiproliferative activity of BT474 (PIK3CA-wild-type, left) and MDA-MB-361 (PIK3CA-mutant, right) cells treated with DMSO (black), combination of fulvestrant (100 nM) and lapatinib (100 nM) (blue), and the triple-combination of fulvestrant, lapatinib, and ipatasertib (1000 nM) (red) for 0, 2, 4, and 8 days (mean ± SD [n = 3]). Relative ATP amounts were compared with the chemiluminescence value of DMSO treatment on day 0. (**b**) Comparison of the inhibitory effect in each drug treatment between BT474 and MDA-MB-361 cell lines. Differences on day8 were analyzed using Student’s t-test. The data represent the mean ± SD (n = 3). (**c**) Representative images of crystal violet staining in BT474 or MDA-MB-361 cells treated with DMSO, the combination of fulvestrant (100 nM) and lapatinib (100 nM), and the triple-combination of fulvestrant, lapatinib, and ipatasertib (1000 nM) for 8 days. (**d**) Quantified data from Fig. 4c. Crystal violet absorbance indicating the amount of normalized protein was measured with a microplate reader and compared with a DMSO control. The data represent mean ± SD (n = 3). Crystal violet absorbance was statistically analyzed using Student’s t-test to compare the double-combination and the triple-combination treatments. Differences were considered significant at *p* ≤ 0.05 (*). (**e**) Effect of combined fulvestrant, lapatinib, and ipatasertib treatment on the apoptosis of BT474 (left) and MDA-MB-361 (right) cells. The cells were treated with DMSO (black), the combination of fulvestrant (100 nM) and lapatinib (100 nM) (blue), and the triple-combination of fulvestrant, lapatinib, and ipatasertib (1000 nM) (red) for 24 h (mean ± SD [n = 3]). Relative caspase-3/7 activities were calculated based on luminescence compared with the DMSO control. Caspase-3/7 activities were analyzed using Student’s t-test to compare the DMSO treatment and the double-combination treatment, and the double-combination and the triple-combination treatments. Differences were considered significant at *p* ≤ 0.05 (*). F, Fulvestrant; L, Lapatinib; I, Ipatasertib; n.s., not significant. (**f**) Representative images of 3D culture in BT474 (left) or MDA-MB-361 (right) cells. (**g**) Antiproliferative activity of 3D-cultured BT474 (PIK3CA-wild-type, left) and MDA-MB-361 (PIK3CA-mutant, right) cells treated with DMSO (black), combination of fulvestrant (100 nM) and lapatinib (100 nM) (blue), and the triple-combination of fulvestrant, lapatinib, and ipatasertib (1000 nM) (red) for 0, 2, 4, and 8 days (mean ± SD [n = 3]). Relative ATP amounts were compared with the chemiluminescence value of DMSO treatment on day 0.

Next, we determined whether pharmacological inhibition of the PI3K pathway would enhance the antiproliferative effects of lapatinib in *PIK3CA*-mut ER+/HER2+ breast cancer cell lines. For this, BT474 and MDA-MB-361 cells were treated with fulvestrant, lapatinib, and ipatasertib alone or in various combinations for 0, 2, 4, and 8 days (Supplementary Fig. 5). Combination treatment with fulvestrant and lapatinib inhibited the proliferation of BT474 cells in a time-dependent manner (Fig. 4a, left panel, blue line); however, addition of ipatasertib had little impact when a triple combination was used (Fig. 4a, left panel, red line). In contrast, addition of ipatasertib to the triple combination markedly decreased cell viability and led to cellular regression in MDA-MB-361 cells compared with those treated with the double combination of fulvestrant and lapatinib (Fig. 4a right panel, blue vs. red lines). Comparison of the effect in each drug treatment between BT474 and MDA-MB-361 cell lines (Fig. 4b) also revealed that the triple combination induced a highly-potent anti-proliferative effects in MDA-MB-361 cells, equivalently to, or even more than, the anti-proliferative effects in the double- or the triple-combination in BT474 cells (Fig. 4b). The results of the crystal violet assay also demonstrated that the triple combination inhibited the proliferation of MDA-MB-361 cells, overcoming the limited antiproliferative effects observed following treatment with the double combination (Fig. 4c,d, and supplementary Fig. 6a,b). Consistent with these results, the effects of ipatasertib on apoptosis as part of triple therapy were also observed in these cell lines. The caspase-3/7 assay revealed that the double combination with fulvestrant and lapatinib significantly elevated caspase-3/7 activity in BT474 cells (Fig. 4e, left, black vs. blue bars), while not significantly in MDA-MB-361 cells (Fig. 4e, right panel, black vs. blue bars). In contrast, addition of ipatasertib to the triple combination markedly significantly elevated caspase-3/7 activity in MDA-MB-361 cells (Fig. 4e, right panel, blue vs. red bars, supplementary Fig. 6c, right panel), while exhibited no significant change in BT474 cells (Fig. 4e, left panel, blue vs. red bars, supplementary Fig. 6c, left panel). Additionally, we conducted antiproliferation studies in a 3D culture system (Fig. 4f), which also demonstrated the potent antiproliferation in treatment with the triple combination in MDA-MB-361 cells, overcoming the limited antiproliferative effects with the double combination (Fig. 4g, supplementary Fig. 5c,d). These results suggest that combination treatment with lapatinib and ipatasertib could be effective against *PIK3CA*-mutant HER2+ breast cancers, overcoming the therapeutic difficulty in the currently-used cancer drugs against these cancers.

**Figure 5:**
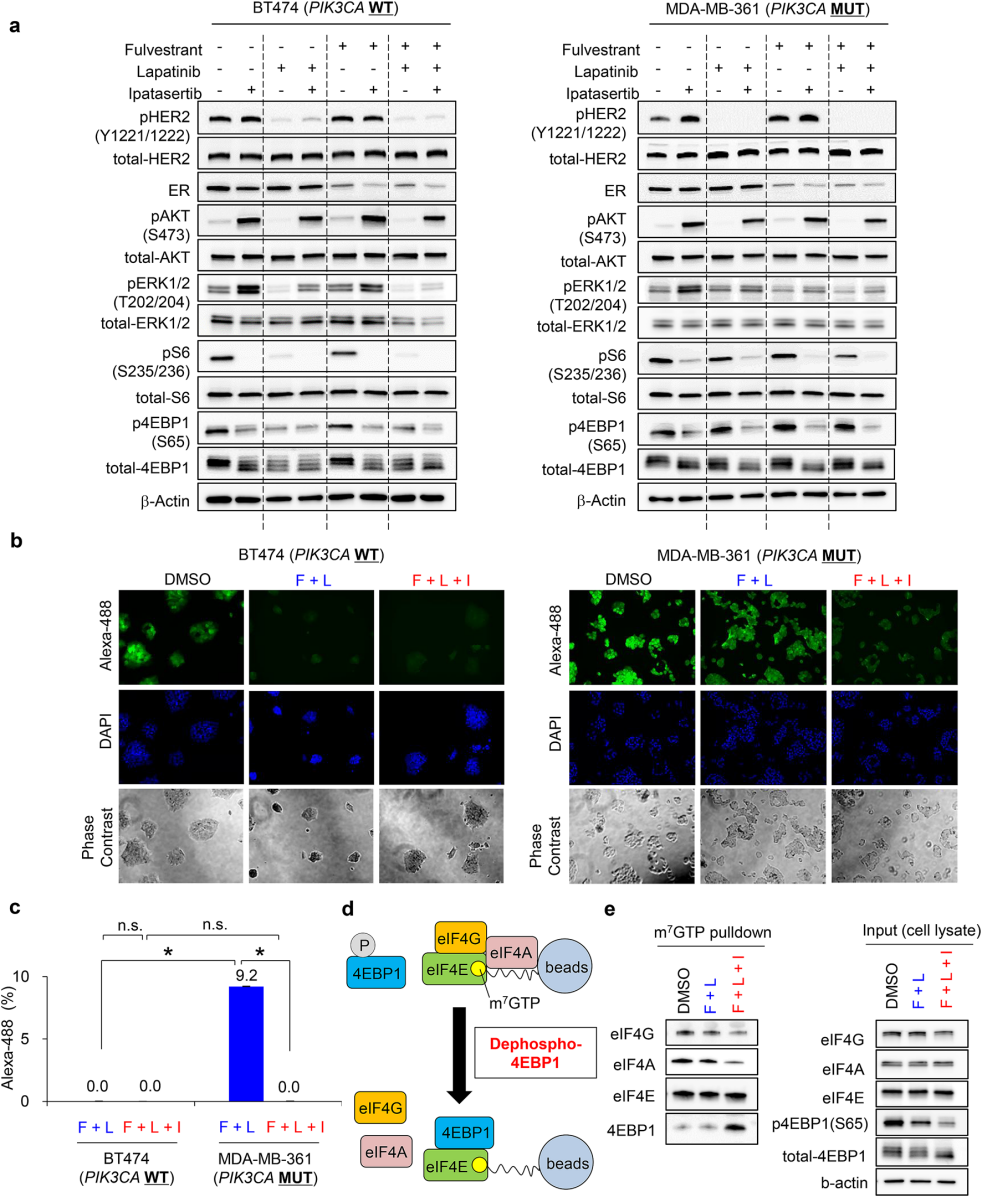
Ipatasertib potently suppresses 4EBP1 phosphorylation in MDA-MB-361 cells treated with combined fulvestrant and lapatinib, inhibiting newly-synthesized proteins. (**a**) Inhibitory effects of ipatasertib on 4EBP1 phosphorylation in fulvestrant and lapatinib-treated BT474 (left) and MDA-MB-361 (right) cells. The cells were treated with fulvestrant (100 nM), lapatinib (100 nM), ipatasertib (1000 nM), and their combinations for 24 h. Plus (+) and minus (−) indicate the presence or absence of treatment. Phosphorylated HER2, ER, and AKT were used as pharmacodynamic markers to confirm target engagement of lapatinib, fulvestrant, and ipatasertib, respectively. β-actin was used as a loading control. Full-length blots are presented in Supplementary Figs. 8 and 9. (**b**) Newly synthesized protein in cells treated with DMSO, combination treatment with fulvestrant and lapatinib, and triple-combination treatment with fulvestrant, lapatinib, and ipatasertib. BT474 (left) and MDA-MB-361 (right) cells were treated with the indicated inhibitors for 24 h, and then Click-iT HPG was incorporated into drug-treated cells for 15 h. Green indicates the newly synthesized proteins detected by the Click-iT assay (Life Technologies) and DAPI-stained nuclei, respectively. (**c**) Quantified data from Fig. 5b. The fluorescent images were captured by BZ-X800 (Keyence Corporation), and the captured images were quantified by a BZ-X800 Analyzer (Keyence Corporation). Alexa-488 fluorescence was normalized using DAPI fluorescence. The y axis indicates the relative Alexa-488 fluorescence, calculated based on fluorescence compared with the DMSO-treatment control. The data represent the mean ± SD (n = 3). The relative Alexa-488 fluorescence was statistically analyzed using Student’s t-test to compare the double-combination and the triple-combination. Differences were considered significant at *p* ≤ 0.05 (*). F, Fulvestrant; L, Lapatinib; I, Ipatasertib; n.s., not significant. (**d**) Experimental schemes of cap-pulldown assay. (**e**) Cap pulldown assay in MDA-MB-361 cells in the triple combination. The cells were treated with fulvestrant (100 nM), lapatinib (100 nM), ipatasertib (1000 nM), and their combinations for 24 h. Immunoblotting of eIF4G, eIF4A, eIF4E, and 4EBP1 were performed in the m7 GTP pulldown samples. eIF4E was used as a pulldown positive control. Immunoblotting of eIF4G, eIF4A, eIF4E, p4EBP1, and 4EBP1 in cell lysates was performed as input controls. β-actin was used as a loading control. Full-length blots are presented in Supplementary Fig. 10.

Next, we examined the signaling pathways downstream of HER2 in the *PIK3CA*-wild-type or the *PIK3CA*-mutant HER2+ breast cancer cell lines treated with each inhibitor alone or in combination. As reported previously^30^, our studies also revealed that ipatasertib notably elevated phosphorylated AKT at Ser-473 (pAKT) in a dose-dependent manner in both BT474 and MDA-MB-361 cells (Fig. 5a and Supplementary Fig. 7). This paradoxical observation may be explained by ipatasertib binding to the active site in AKT, subsequently protecting these sites from phosphatases and increasing AKT phosphorylation^21,31^, while the downstream of Akt pathway is expected to be suppressed in treatment with ipatasertib^30^. Therefore, AKT phosphorylation does not reflect its activation. In BT474 cells, lapatinib alone or in combination with fulvestrant potently inhibited both pERK and p4EBP1, and no additional effect of ipatasertib was detected (Fig. 5a, left panel, lane 3, 7 and lane 8). In contrast, in MDA-MB-361 cells, lapatinib treatment as a single-agent or in combination with fulvestrant potently inhibited pERK; however, a substantial amount of p4EBP1 was still detected (Fig. 5a, right panel, lane 3 and lane 7). Furthermore, addition of ipatasertib to lapatinib and fulvestrant markedly decreased p4EBP1 expression (Fig. 5a, right panel, lane 7 and lane 8). Results of the Click-iT assay also revealed that, although signals for newly-synthesized proteins were detected in MDA-MB-361 cells treated with the double combination, ipatasertib treatment as part of triple therapy reduced these signals to the detection limit (Fig. 5b,c). In BT474 cells, combination treatment with fulvestrant and lapatinib effectively inhibited the synthesis of new proteins (Fig. 5b,c). Dephosphorylated 4EBP1 binds to eIF4E to inhibit the complex formation of eIF4E–eIF4G–eIF4A subunits, and suppresses cap-dependent protein translation^32^. Next, to determine the effects of the triple combination treatment on the complex formation of these translation subunits, we performed a pulldown assay with a γ-aminophenyl-7-methyl guanosine (m^7^ GTP) C_10_-spacer agarose beads in MDA-MB-361 cell lysate with the indicated combination treatments (Fig. 5d). The pulldown assay revealed that the triple combination treatment recruited 4EBP1 to the m^7^ GTP-eIF4E cap-component, replacing the eIF4E–eIF4G–eIF4A complex with the eIF4E–4EBP1 complex (Fig. 5e, left). Dephosphorylation of 4EBP1 in the triple treatment was confirmed by immunoblotting in the control cell lysate (Fig. 5e, right).

**Figure 6:**
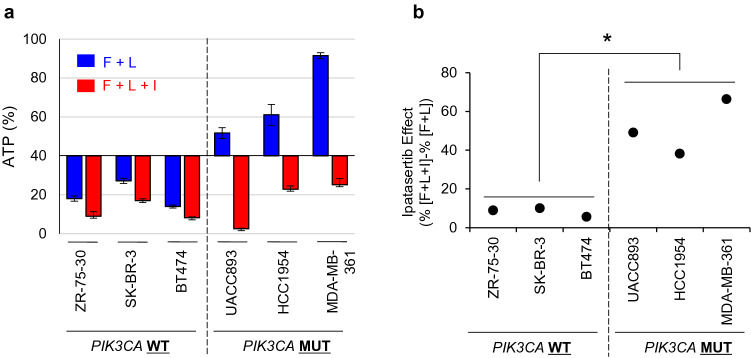
Combination treatment with lapatinib and ipatasertib enhances antiproliferative activity in *PIK3CA*-mutant HER2+ breast cancer cell lines. (**a**) Antiproliferative activity of combination treatment with fulvestrant and lapatinib, and triple-combination treatment with fulvestrant, lapatinib, and ipatasertib in *PIK3CA*-wild-type and *PIK3CA*-mutant HER2+ breast cancer cell lines. Three PIK3CA-wild-type (BT474, SK-BR-3, and ZR-75-30; left) and three PIK3CA-mutant (UACC893, HCC1954, and MDA-MB-361; right) HER2+ breast cancer cell lines were used. Cells were treated with fulvestrant and lapatinib (blue), or triple-combination treatment with fulvestrant, lapatinib, and ipatasertib (red) for 8 days (mean ± SD [n = 3]). The Y-axis indicates the relative ATP amounts (%). The relative ATP amounts were calculated using chemiluminescence assay and compared with the chemiluminescence of the DMSO control on day 8. (**b**) Quantified data of ipatasertib combination effects form Fig. 6a. The effects of ipatasertib were calculated using the following formula: ipatasertib combination effects = (%ATP with triple-combination treatment with fulvestrant, lapatinib, and ipatasertib)—(%ATP with fulvestrant and lapatinib treatment). Statistical analyses were performed using the non-parametric Wilcoxon–Mann–Whitney test. Differences were considered significant at *p* < 0.05. F, Fulvestrant; L, Lapatinib; I, Ipatasertib.

To confirm the effects of combined ipatasertib and lapatinib in *PIK3CA*-mut/ER-/HER2+ cells, additional cell lines were used as described above (Fig. 3c). The results of the cell viability assay revealed that, although the antiproliferative effect of lapatinib alone was limited in *PIK3CA*-mutant cell lines compared with the *PIK3CA*-wild-type cell lines (Fig. 6a, blue bars), ipatasertib enhanced the antiproliferative effect induced by lapatinib in the *PIK3CA*-mutant cell lines more prominently compared to the *PIK3CA*-wild-type cell lines (Fig. 6a,b). Taken together, these results suggest that ipatasertib treatment in combination with lapatinib has potential to overcome resistance HER2 therapy in patients with *PIK3CA*-mutant HER2+ breast cancers.

### Inhibitors targeting the PI3K pathway enhance the antiproliferative activity of lapatinib in the PIK3CA-mut HER2+ breast cancer cell line

**Figure 7:**
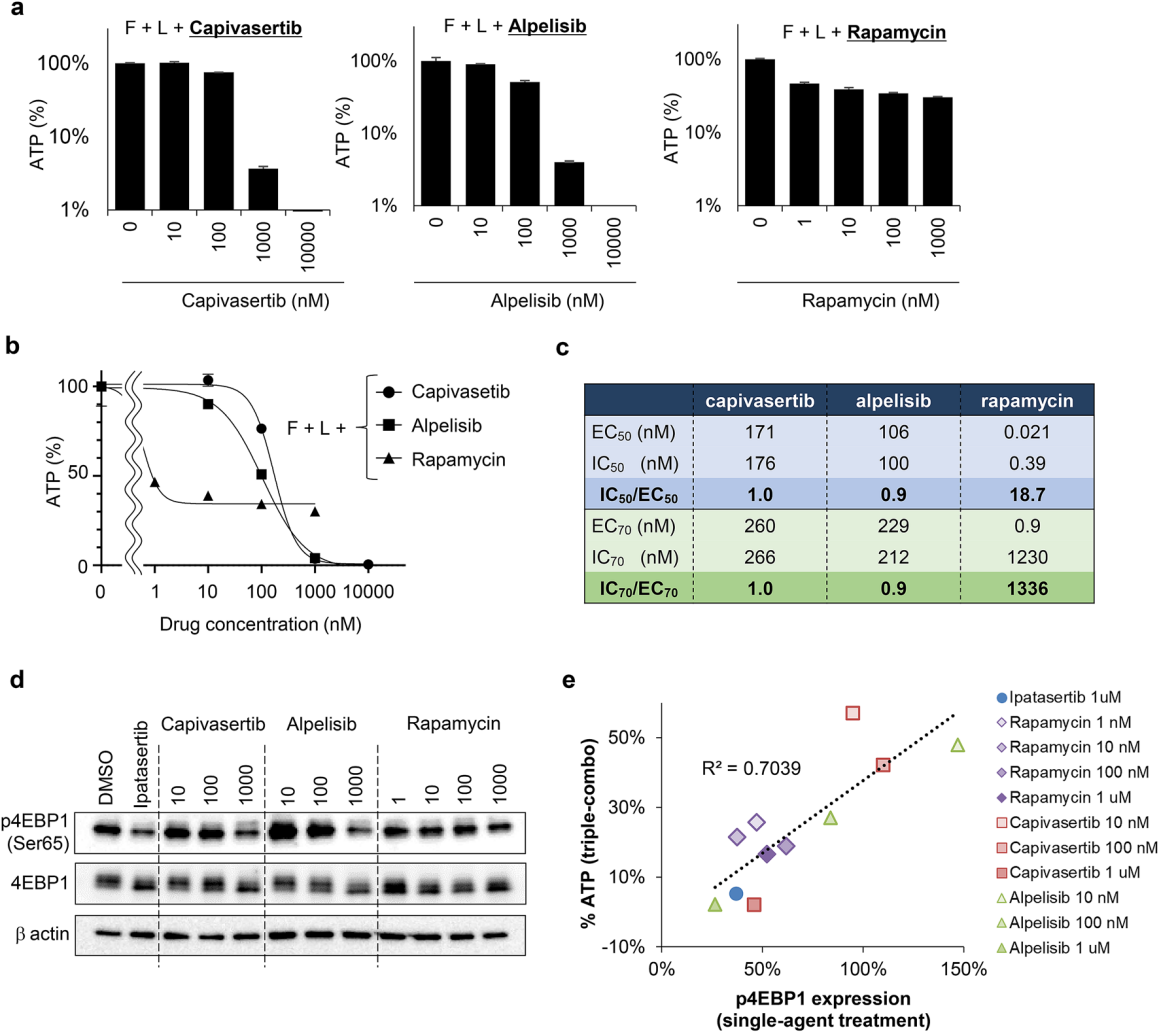
Phosphorylated 4EBP1 is a potential predictive biomarker for combination treatment with lapatinib and ipatasertib. (**a**) Antiproliferative activity of combination treatment with fulvestrant (100 nM), lapatinib (100 nM), and PI3K pathway inhibitors (capivasertib, alpelisib, and rapamycin) in MDA-MB-361 (*PIK3CA*-mutant) cells. The cells were treated for 8 days. Relative ATP amounts were calculated using a chemiluminescence assay and compared with the chemiluminescence value of combination treatment with fulvestrant and lapatinib on day 8 (mean ± SD [n = 3]). (**b**, **c**) Dose response curve and EC/IC of PI3K pathway inhibitors (capivasertib, alpelisib, and rapamycin) in combination with fulvestrant (100 nM) and lapatinib (100 nM). This graph and calculation was performed by GraphPad Prism. (**d**) Inhibitory effects of Capivasertib, alpelisib, and rapamycin on 4EBP1 phosphorylation in MDA-MB-361 cells. Cells were treated with capivasertib, alpelisib, and rapamycin at the indicated concentrations for 24 h. β-actin was used as a loading control. Full-length blots are presented in Supplementary Fig. 11b. (**e**) Correlation between p4EBP1 expression and antiproliferative activity of triple treatment combinations in MDA-MB-361 cells. Cells were treated with the triple-combination of fulvestrant, lapatinib, and ipatasertib (blue), rapamycin (purple), capivasertib (red), or alpelisib (green) at the indicated concentrations for 8 days (n = 3). The X-axis indicates p4EBP1 expression in cells treated with the indicated PI3K inhibitors as monotherapy for 24 h. The Y-axis indicates relative ATP amounts (%) in the triple combinations. The relative ATP amounts were calculated using a chemiluminescence assay and compared with the chemiluminescence value of the DMSO control on day 8. p4EBP1 expression was calculated using immunoblotting data quantified by ImageJ software. F, Fulvestrant; L, Lapatinib.

Studies investigating combination treatment with lapatinib and ipatasertib in the PIK3CA-mut HER2+ breast cancer cell lines revealed that inhibition of both the MAPK and PI3K pathways is critical to inhibit proliferation in HER2+ breast cancer cell lines, and that expression levels of p4EBP1 were correlated with the antiproliferative effects of the inhibitors (Figs. 3a, 5a). To validate these findings, we also performed the combination studies using other chemical inhibitors targeting PI3K pathway: capivasertib, alpelisib, and rapamycin were used as AKT, PI3K, and mTOR inhibitors, respectively^33^. As a control, ipatasertib treatment at 1 μM was used in the following experiments. MDA-MB-361 cells were treated with capivasertib, alpelisib, or rapamycin at the indicated concentrations in the presence of fulvestrant and lapatinib. Triple therapy with one of these PI3K inhibitors also enhanced the antiproliferative activities of fulvestrant and lapatinib in a dose-dependent manner (Fig. 7a and Supplementary 11a), while the combination effects with rapamycin (Fig. 7a, right panel) was limited compared to those with capivasertib or alpelisib (Fig. 7a, left and middle panels). We next calculated 50% and 70% effective concentration (EC_50_ and EC_70_), and 50% and 70% inhibitory concentration (IC_50_ and IC_70_) based on the dose–response curve in each treatment (Fig. 7b,c). The EC and IC values were used as indexes for drug-effectiveness and antiproliferation, respectively. The ratios of IC_50_/EC_50_ and IC_70_/EC_70_ in capivasertib treatment and those in alpelisib treatment were 1.0, 1.0, 0.9, and 0.9, respectively (Fig. 7c). These results indicate that the drug-effectiveness of these compounds would be a key driver of antiproliferation in the triple-combination treatment. In rapamycin treatment, on the contrary, the ratios of IC_50_/EC_50_ and IC_70_/EC_70_ were 18.7 and 1336, showing an enormous gap between drug-effectiveness and antiproliferation. Immunoblotting also revealed that treatment with capivasertib or alpelisib markedly decreased p4EBP1 expression in a dose-dependent manner, although treatment with rapamycin caused the inhibitory effect of p4EBP1 to plateau at 1–1000 nM, and large amounts of p4EBP1 could still be detected at 1000 nM (Fig. 7d). Given that rapamycin could partially, but not profoundly, inhibits mTORC1^34–36^, which is an upstream regulator of 4EBP1 phosphorylation, leaking of p4EBP1 may be still observed even in the plateau concentration of rapamycin drug-effectiveness. Comparison analysis between p4EBP1 and ATP amount in MDA-MB-361 cells revealed that the single-agent inhibitory effects of these inhibitors on p4EBP1 significantly correlated with antiproliferative effects in triple-combination (Fig. 7e). These results suggest that dynamics of expression level of p4EBP1 in response to the drug treatment may be used as a biomarker against currently-used anti-HER2 therapies, and as an efficacious-linking biomarker of these combination.

## Discussion

In this study, we demonstrated that *PIK3CA* mutations, which lead to constitutive activation of the PI3K/AKT/mTOR pathway, play important roles in resistance against HER2 monotherapy in breast cancer cell lines. We also demonstrated that combination therapy with a HER2 inhibitor and a AKT inhibitor, as well as other PI3K/AKT/mTOR pathway inhibitors, could overcome the therapeutic limitations associated with anti-HER2 monotherapy in the *PIK3CA*-mutant HER2+ breast cancers. Furthermore, following combination treatment, p4EBP1 expression was correlated with the antiproliferative activities, suggesting that p4EBP1 has potential as a diagnostic and/or efficacy-linking biomarkers for these combination therapies.

Although a number of preclinical studies have supported involvement of the PI3K/AKT/mTOR pathway in resistance to anti-HER2 therapies^7^, the clinical significance of the PI3K/AKT/mTOR pathway in the mechanisms of resistance against anti-HER2 therapies remains inconclusive. For example, in the phase III CLEOPATRA study of anti-HER2 antibody therapies, patients with *PIK3CA*-mutant tumors had a worse prognosis than those with *PIK3CA*-wild-type tumors^13^*.* In contrast, other clinical studies, TRYPHAENA or NeoSphere, demonstrated that *PIK3CA* mutations were not significantly associated with resistance to anti-HER2 antibody therapies^10,12^. To re-evaluate the clinical impacts of the PI3K/AKT/mTOR pathway on anti-HER2 therapy pathway, we conducted a retrospective analysis using clinical data from patients with HER2+ breast cancer who had received anti-HER2 therapies (Fig. 1). Supporting the findings of the CLEOPATRA study, we demonstrated that mutations in the PI3K/AKT/mTOR pathway are associated with poor overall survival following anti-HER2 therapies in patients with HER2+ breast cancer. These findings suggest that active mutations in the PI3K/AKT/mTOR pathway may act as strong negative prognostic biomarkers for the identification of patients with HER2+ breast cancer that have a high-unmet need for effective therapies.

Several small-molecule inhibitors targeting the PI3K/AKT/mTOR pathway are being developed as cancer therapeutics, including pan-PI3K inhibitors, isoform-specific PI3K inhibitors, dual PI3K/mTOR inhibitors, mTORC1 inhibitors, dual mTORC1/2 inhibitors, and AKT inhibitors^17^. Because the single-agent activities of these inhibitors have been modest in clinical trials, combination therapies with currently-used cancer drugs have demonstrated beneficial effects for the PI3K/AKT/mTOR inhibitors^37,38^; anti-HER2 therapies represent promising combination partners. Preclinical studies have demonstrated that combination treatment with PI3K/AKT/mTOR pathway inhibitors enhances the antiproliferative activities of anti-HER2 therapies, overcoming resistance to single-agent treatment with anti-HER2 therapies^39–43^. Based on preclinical evidence, clinical trials are underway to investigate combination therapies^19,20^. Small-molecule inhibitors targeting AKT have recently attracted attention as a new class of PI3K/AKT/mTOR pathway inhibitors, which possess a wide variety of chemical structures, isoform selectivity, and enzymatic modes of action (for example, ATP-competitive, allosteric, or irreversible inhibitors)^44^. In preclinical studies, combination treatment with a nucleotide-derivative AKT inhibitor significantly enhanced the antiproliferative activity of an anti-HER2 antibody in breast cancer xenograft models, while combination treatment with a rapamycin-derivative mTOR inhibitor did not^45^. Accordingly, our studies also revealed that the ATP-competitive AKT inhibitor ipatasertib exhibited antiproliferative effects when used in combination with the HER2 inhibitor lapatinib in the HER2+ *PIK3CA*-mutant breast cancer cell line, while the combination effects with rapamycin were less pronounced. These findings indicate that AKT inhibitors may have more potential in combinational therapies with anti-HER2 therapies compared with the rapamycin-derivative mTOR inhibitors, which did not demonstrate clinically-significant benefit in combination with anti-HER2 therapies^20,46,47^. Given that their clinical efficacy and tolerability were confirmed in HER2-negative breast cancer^22^, AKT inhibitors, such as ipatasertib, may be evaluated in combination with anti-HER2 therapies targeting patients with HER2+ breast cancer with constitutive activation of the PI3K/AKT/mTOR pathway.

During the development of targeted therapies, identification of specific biomarkers able to predict drug efficacy is essential. In this study, we demonstrate that a HER2 inhibitor insufficiently suppresses p4EBP1 as well as pS6 in *PIK3CA*-mutant HER2+ breast cancer cell lines (Fig. 3a), and that these cells are resistant to the HER2 inhibitor. p4EBP1 is a downstream target in the PI3K/AKT/mTOR pathway, and its expression appears to reflect the activation status of the PI3K/AKT/mTOR pathway. In addition, p4EBP1 is upregulated by a variety of gene modifications, such as *PIK3CA* mutations, PTEN loss, AKT mutation, suggesting that p4EBP1 may have potential as a biomarker for each mutation status. Furthermore, expression of p4EBP1 following treatment with AKT inhibitor was well-correlated with antiproliferative activities. Thus, p4EBP1 has a potential as: (1) an “exclusive biomarker” for anti-HER2 therapy as a single cell signaling modifying strategy; (2) a “diagnostic biomarker” to recommend the combination therapy with the PI3K/AKT/mTOR pathway inhibitors; and (3) an “efficacy-linking biomarker” to predict the response to drug treatment. Further preclinical and clinical studies need to be conducted to clarify more precisely the potential of p4EBP1 as biomarkers, which would shed light on the next generation of HER2 therapy.

In summary, we demonstrated that the PI3K/AKT/mTOR pathway plays an important role in resistance of breast cancer cells to single-agent HER2 therapy, and combination therapy with HER2 and PI3K/AKT/mTOR inhibitors could overcome the PI3K/AKT/mTOR pathway-mediated resistant mechanism in HER2+ breast cancers. Furthermore, p4EBP1 has potential as a diagnostic and/or efficacy-linking biomarker for these combination therapies. Further investigations into mechanisms underlying resistance to anti-HER2 therapy in the PI3K/AKT/mTOR pathway will provide important insights into optimal clinical strategies for combination therapy.

## Methods

### Compounds

Lapatinib (SYN-1052), ipatasertib (18412), fulvestrant (F1144), capivasertib (S8019), rapamycin (553211), alpelisib (A-4477) were purchased from SYNkinase (Parkville, Australia), Cayman Chemical (Ann Arbor, MI, USA), and Tokyo Chemical Industry (Tokyo, Japan), Selleck (Houston, USA), Merk (Darmstadt, Germany), and LC laboratories (Massachusetts, USA), respectively.

### Cell lines and culture

Human breast cancer cell lines, BT474, MDA-MB-361, SK-BR-3, ZR-75-30, UACC893, and HCC1954, were obtained from American Type Culture Collection (Manassas, VA, USA). The cells were cultured at 37 °C with 5% CO_2_ in RPMI-1640 medium (Sigma-Aldrich, St. Luis, MO) supplemented with 10% fetal bovine serum (S1610-500, Biowest, Nuaillé, France) and 1% penicillin/streptomycin (15140-122, Thermo Fisher Scientific, MA, USA).

### Antibodies

The following anti-human antibodies were used for immunoblotting: anti-phosphorylated Akt at Ser-473 (#4060, Cell Signaling Technology), anti-Akt (#4685, Cell Signaling Technology), anti-phosphorylated Erk1/2 at Thr202/Thy204 (#9106, Cell Signaling Technology), anti-Erk1/2 (#9102, Cell Signaling Technology), anti-phosphorylated 4EBP1 at Ser-65 (#9451, Cell Signaling Technology), anti-ER (#13258, Cell Signaling Technology), anti-4EBP1 (#9452, Cell Signaling Technology), anti-phosphorylated HER2 at Tyr-1221/1222 (#2243, Cell Signaling Technology), anti-HER2 (#2248, Cell Signaling Technology), anti-eIF4G (#2498, Cell Signaling Technology), anti-eIF4E (#9742, Cell Signaling Technology), anti-eIF4A (#2013, Cell Signaling Technology), and anti-β-actin (#4970, Cell Signaling Technology).

### Cell growth assay (CellTiter-Glo luminescent cell viability assay)

Cell growth was evaluated based on intracellular ATP concentrations using the CellTiter-Glo luminescent cell viability assay (Promega Corp., Madison, WI, USA). Approximately 7500 UACC893 and ZR75-30 cells; 5000 MDA-MB-361 cells; 2500 BT474 and SK-BR-3 cells; and 1250 HCC1954 cells per well were seeded into 96-well plates with 50 μL RPMI medium, and allowed to adhere to the plates for at least 16 h. Then, fulvestrant, lapatinib, ipatasertib, the double-combinations, or the triple-combination were added to each well with 150 μL RPMI medium to reach the indicated concentrations and cells were incubated for a further 0, 2, 4, and 8 days. The CellTiter-Glo 2.0 luminescent cell viability assay was performed according to the manufacturer’s protocol. Luminescence was measured with a SpectraMax L luminescence microplate reader (Molecular Devices, Sunnyvale, CA, USA). Relative ATP levels were calculated based on luminescence compared with a control (DMSO treatment).

### Cell viability assay (crystal violet staining)

Cell viability was evaluated using crystal violet staining (V5265, Sigma). Approximately 20,000 BT474 and MDA-MB-361 cells were seeded into 24-well plates with 500 μL RPMI medium, and allowed to adhere to the plates for at least 16 h. The cells were treated with fulvestrant (100 nM), lapatinib (100 nM), ipatasertib (1000 nM), the double-combinations, or the triple-combination for 10 days. The cells were fixed with 4% paraformaldehyde for 10 min at room temperature (RT), and methanol at − 20 °C for 5 min. The fixed cells were then stained with 0.5% crystal violet solution for 30 min. After washing with water, cellular crystal violet was dissolved using a buffer containing 30% ethanol and 1% acetic acid. The absorbance of dissolved crystal violet was measured with a microplate reader at 590 nm. Relative protein levels were calculated on the basis of absorbance in comparison with those of the control (DMSO treatment).

### Apoptosis assay (Caspase-3/7-Glo luminescent assay)

Apoptosis was evaluated based on Caspase-3/7 activity using a Caspase-3/7-Glo luminescent kit (Promega). Approximately 5000 MDA-MB-361 cells, and 2500 BT474 cells were seeded into 96-well plates in 50 μL RPMI medium, and allowed to adhere to the plates for at least 16 h. Then, fulvestrant, lapatinib, ipatasertib, the double-combinations, or the triple-combination were added to each plate with 150 μL RPMI medium to reach the indicated concentrations, and plates were incubated for a further 4 days. The Caspase-3/7-Glo luminescent assay was performed according to the manufacturer’s protocol. Chemical luminescence was measured using a microplate reader, as described above. Relative caspase-3/7 activities were calculated on the basis of luminescence compared with that of the control (DMSO treatment).

### 3D Cell growth assay

NanoCulture 96-well Plate was used for 3D culture (Cat#: NCP-LH-96, ORGANOGENIX, Kanagawa, Japan). BT474 and MDA-MB-361 cells were seeded at the density of 7500 cells/well and 10,000 cells/well in the 96-well plates with 50 μL RPMI medium, respectively. The spheroid formations in these cells were confirmed by microscope, and then the spheroid cells were treated with the drugs at the indicated concentrations in 200 μL RPMI medium for 0, 2, 4, and 8 days. The CellTiter-Glo 2.0 luminescent cell viability assay was performed according to the manufacturer’s protocol. Luminescence was measured with a SpectraMax L luminescence microplate reader (Molecular Devices, Sunnyvale, CA, USA). Relative ATP levels were calculated based on luminescence compared with the DMSO-treatment control.

### Immunoblotting

The cells were lysed in Cell Lysis Buffer (#9803, Cell Signaling Technology) containing PMSF (022-15371, Wako) and protease inhibitors (P8340, Sigma). The cell lysates were suspended in Lane Marker Reducing Sample Buffer (Cat#39000, Thermo Scientific) for protein concentrations of 1–2 mg/mL, and then boiled at 95 °C for 5 min. Five–ten-micrograms of protein were electrophoresed in 4–20% SDS-PAGE gels (Bio-Rad, CA) and transferred onto a PVDF membrane (Millipore, MA). After blocking with 4% bovine serum albumin (10-735-108-001, Roche), the membrane was incubated with the primary antibodies (1:1000 dilution) followed by incubation with horse radish peroxidase-conjugated secondary antibodies against anti-mouse IgG (#7076, Cell Signaling Technology) or anti-rabbit IgG (#7074, Cell Signaling Technology) (1:5000 dilution). The immunoblotted proteins were visualized by luminol-based enhanced chemiluminescence (RPN2232, GE healthcare), and the luminescent images were captured by ImageQuant LAS 4010 (GE healthcare). We processed images of electrophoretic gels and blots according to the digital image and integrity policies of Scientific Reports.

### Detection of newly synthesized proteins

Newly synthesized proteins were detected using Click-iT HPG Alexa Fluor Protein Synthesis Assay Kits (C10428, Life Technologies). Approximately 25,000 BT474 cells and 30,000 MDA-MB-361 cells were seeded into 96-well plates with 100 μL RPMI medium, and allowed to adhere to the plates for 24 h. The cells were treated with fulvestrant at 100 nM, lapatinib at 100 nM, ipatasertib at 1000 nM, the double-combinations, or the triple-combination for 24 h, and then Click-iT HPG (l-homopropargylglycine), an amino acid analog of methionine containing an alkyne moiety, was incorporated into drug-treated cells for 15 h. The Click-iT HPG Alexa Fluor Protein Synthesis Assay was performed according to the manufacturer’s protocol. Fluorescent images were captured and quantified by BZ-X800 Analyzer (Keyence Corporation).

### Cap pull-down assay

MDA-MB-361 cells were treated with DMSO, Fulvestrant (100 nM), Lapatinib (100 nM), and their combinations for 24 h, and then were lysed in the lysis buffer (150 mM NaCl, 50 mM Tris–HCl (pH 7.6), 1 mM EDTA and 0.5% NP-40) supplemented with PMSF, protease inhibitors (P8340, SIGMA), and phosphatase inhibitors (160-24371, Wako). The cell lysates were subjected to rotatory incubation at 4 °C, and then the supernatants were collected by spinning-down at 14,000* g* for 15 min. The supernatants were incubated with immobilized γ-Aminophenyl-m7GTP (C10-spacer) agarose beads (AC-155S, Jena Bioscience) on the rotator overnight at 4 °C. After incubation, beads were washed with the lysis buffer three times. Proteins bound to beads were eluted with SDS-PAGE sample buffer (39000, Thermo Fisher Scientific) to be analyzed by immunoblotting.

### Analysis of dose response curve

Creating and analyzing dose response curves were performed by GraphPad Prism (GraphPad Software, Inc., San Diego, CA, USA). EC anything was calculated using the GraphPad QuickCalcs Web site: https://www.graphpad.com/quickcalcs/Ecanything1.cfm (accessed May 2020).

IC anything was calculated according to GraphPad Curve Fitting Guide “Equation: log(inhibitor) vs. normalized response”.

### Survival analysis of HER2+ breast cancer patients

Clinical data of 197 patients treated with anti-HER2 therapy were extracted from the literature by Razavi et al. ^24^. And among those patients, we further extracted and reanalyzed 186 patients diagnosed as HER2-positive breast cancer clinically and pathologically during clinical course (Table 1, Supplementary Table 1). Overall survival (OS) was defined as the duration from the initiation of the first anti-HER2 therapy to death, or the day of the last follow-up. OS was analyzed in patients with HER2+, and in those with HER2+ and ER+ breast cancer (Table 1). Patients were stratified by PI3K pathway status, including *PIK3CA* mutation, *PIK3R1* mutation, *AKT1* mutation, and *PTEN* loss. A log-rank test was used to compare overall survival between the two according to PI3K pathway status. Statistical analyses were performed by GraphPad Prism (GraphPad Software, Inc., San Diego, CA, USA), and differences were considered significant at *p* < 0.05. The patients are not directly involved in the study.

### Statistical analysis

Parametric statistical analysis by Student's t-test or non-parametric statistical analysis by Wilcoxon–Mann–Whitney test were used to compare in vitro antiproliferation data in R version 3.5.0. Differences were considered significant at *p* < 0.05.

## Supplementary Information

Supplementary Information.

## Data Availability

The datasets generated during and/or analysed during the current study are available from the corresponding author on reasonable request.
