# Supplementary material for: Combination treatment with a PI3K/Akt/mTOR pathway inhibitor overcomes resistance to anti-HER2 therapy in PIK3CA-mutant HER2-positive breast cancer cells
Source: Sci Rep. 2020 Dec 10;10:21762. doi: 10.1038/s41598-020-78646-y (PMC7729878; doi:10.1038/s41598-020-78646-y)
Supplement: Supplementary file 1 — Supplementary Information. [file 41598_2020_78646_MOESM1_ESM.pdf]

Supplemental Information

Title: **Combination treatment with a PI3K/Akt/mTOR pathway inhibitor overcomes resistance to anti-HER2 therapy in *PIK3CA*-mutant HER2-positive breast cancer cells**

Yumi Fujimoto[1†], Tomoko Yamamori Morita[2†] Akihiro Ohashi[2], Hiroshi Haeno[2, 3], Yumi Hakozaki[2], Masanori Fujii[2], Yukie Kashima[2], [*]Susumu S. Kobayashi[2,4], and [*]Toru Mukohara[1]

[1]Department of Breast and Medical Oncology, National Cancer Center Hospital East, Kashiwa, Chiba, Japan. [2]Division of Translational Genomics, Exploratory Oncology Research and Clinical Trial Center, National Cancer Center, Kashiwa, Chiba, Japan. [3]Department of Computational Biology and Medical Sciences, Graduate School of Frontier Sciences, The University of Tokyo, Kashiwa, Chiba, Japan. [4]Department of Medicine, Beth Israel Deaconess Medical Center/Harvard Medical School, Boston, MA, USA.

† These authors contributed equally to this work.

Correspondence to:

[*] Susumu Kobayashi, MD, PhD - Division of Translational Genomics, Exploratory Oncology Research and Clinical Trial Center, National Cancer Center, Kashiwa, Chiba, Japan. Tel and Fax: +81-4-7134-8786, Email: sukobaya@east.ncc.go.jp

[*] Toru Mukohara, MD, DMedSci. - Department of Breast and Medical Oncology, National Cancer Center Hospital East, Kashiwa, Chiba, Japan. Tel: +81-4-7133-1111, Fax: +81-4-7134-6922, Email: tmukohar@east.ncc.go.jp

Inventory of Supplemental Information

Supplementary Figure S1. (Related to Figure 1)

Supplementary Figure S2.

Supplementary Figure S3. (Related to Figure 3)

Supplementary Figure S4. (Related to Figure 3)

Supplementary Figure S5. (Related to Figure 4)

Supplementary Figure S6. (Related to Figure 4)

Supplementary Figure S7. (Related to Figure 5)

Supplementary Figure S8. (Related to Figure 5)

Supplementary Figure S9. (Related to Figure 5)

Supplementary Figure S10. (Related to Figure 5)

Supplementary Figure S11. (Related to Figure 7)

Supplementary Table S1. (Related to Figure 1)

Supplementary Figure 1

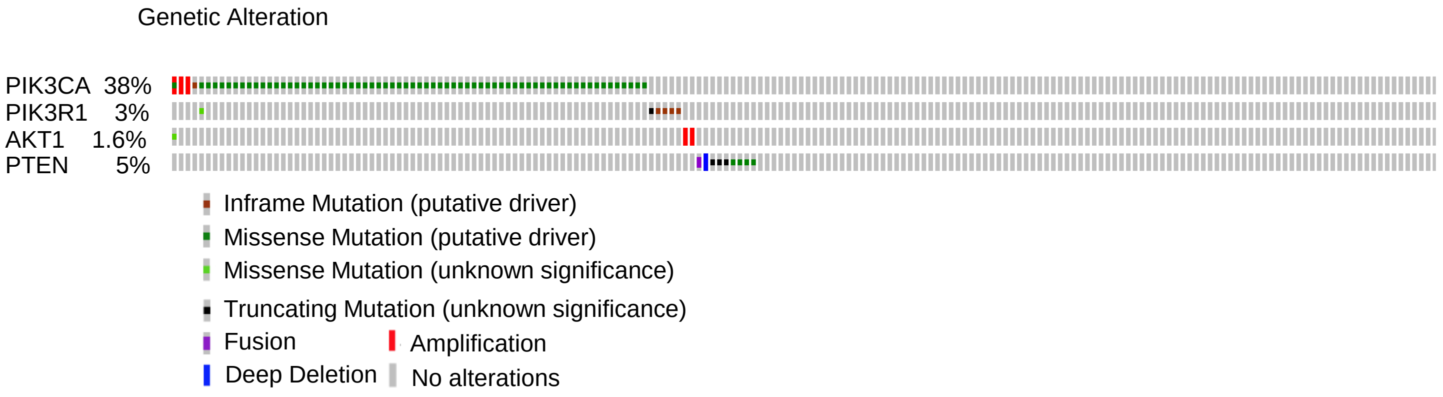

Supplementary Figure 1. Overall survival for patients with HER2-positive and ER negative (HER2+/ER-) breast cancer. (related to main figure 1)

Oncoprint visualization of the PIK3CA, PIK3R1, AKT1, and PTEN alterations in the cohorts tested. This was generated on cBioPortal.org.

Supplementary Figure 2

c.333 G/C

BT474

c.333 G(WT)

MDA-MB-361
(control)

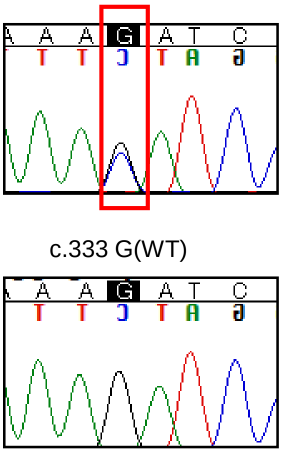

Gene sequence c.333 G>C (Heterozygous)
Protein sequence p.K111N

Supplementary Figure 2. Mutation analysis of PIK3CA gene in BT474 cell line

BT474 cell has heterozygous mutation (c.333 G>C) in PIK3CA gene sequence. Primers (Forward primer : 5'-TGCCTCCGTGAGGCTACATTA- 3' , Reverse primer: 5'-TAGAGCAAAGGCAGCAAACATTC-3') were used for PIK3CA mutation analysis to PCR amplify genomic DNA. MDA-MB-361 cell line was used as a control cell line.

Supplementary Figure 3

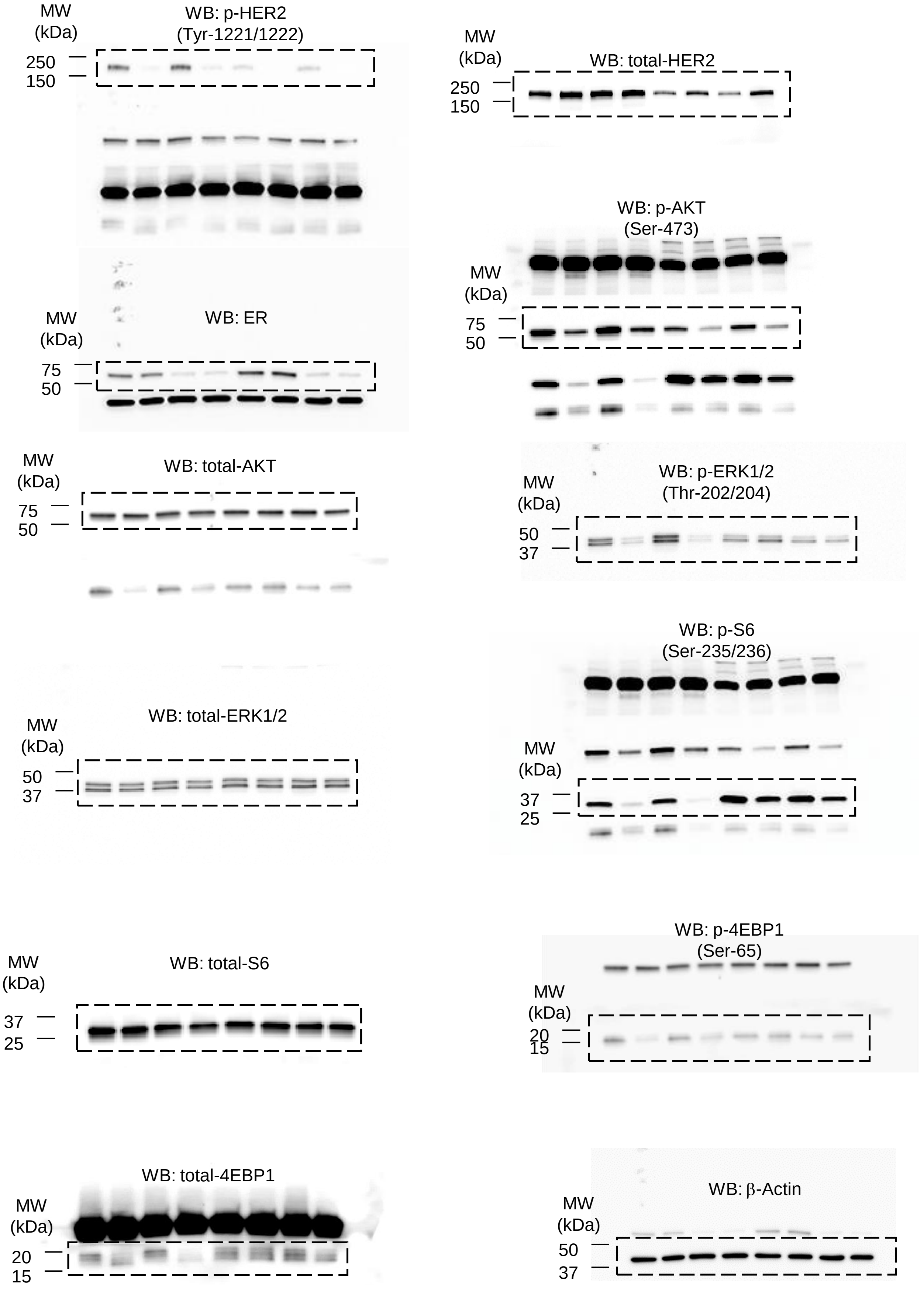

Supplementary Figure 3. Original images of immunoblots for figure. 3a

Supplementary figure 3 related to main figure 3a.

Supplementary Figure 4

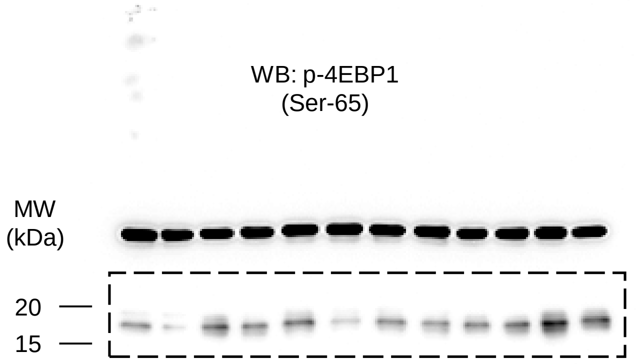

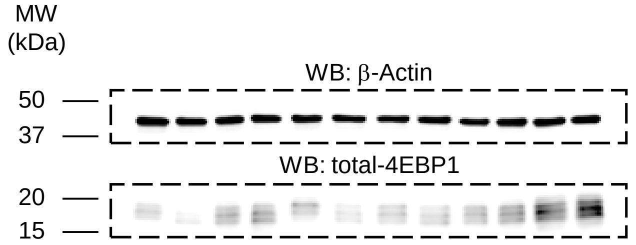

Supplementary Figure 4. Original images of immunoblots for figure. 3e

(related to main figure. 3e)

Supplementary Figure 5

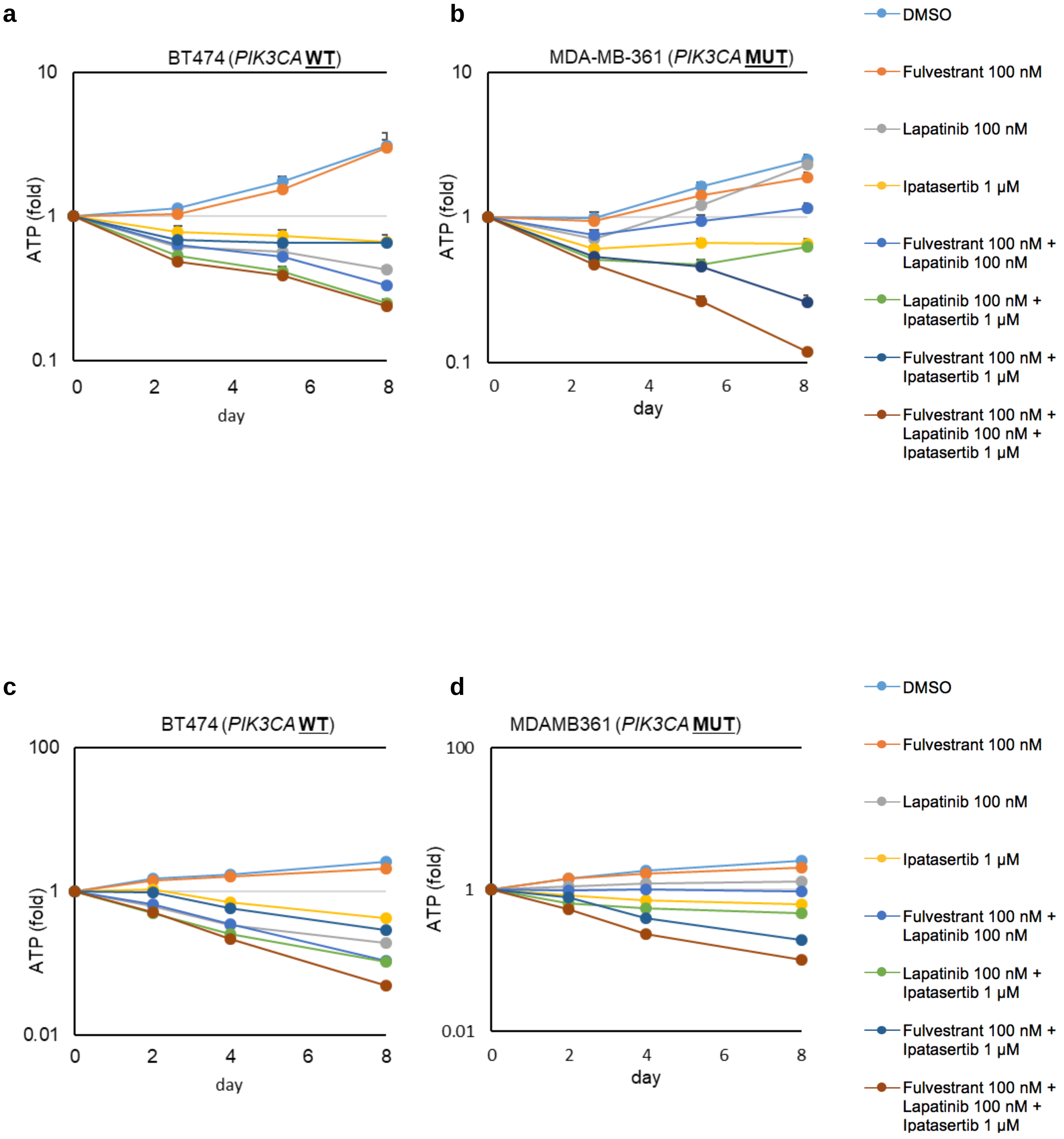

Supplementary Figure 5. Ipatasertib potently enhances antiproliferative activity of fulvestrant and lapatinib combination in a PIK3CA-mutant HER2+/ER+ breast cancer cell line (related to main figure 4a and 4b).

(a) (b) Antiproliferative activity of the combination of fulvestrant, lapatinib, and ipatasertib in BT474 (PIK3CA-wild-type, (a)) and MDA-MB-361 (PIK3CA-mutant, (b)) cells. (c) (d) Antiproliferative activity of 3D-cultured BT474 (PIK3CA-wild-type, (c)) and MDA-MB-361 (PIK3CA-mutant, (d)) cells. The cells were treated with DMSO (light blue), fulvestrant (100 nM, orange), lapatinib (100 nM, gray), ipatasertib (1 μM, yellow), the double-combination of fulvestrant and lapatinib (blue), the double-combination of lapatinib and ipatasertib (green), the double-combination of fulvestrant and ipatasertib (deep blue), and the triple-combination of fulvestrant, lapatinib and ipatasertib (brown) for 0, 2, 4, and 8 days (mean ± SD [n = 3]). Relative ATP amounts were calculated using a chemiluminescence assay and compared with the chemiluminescence value of DMSO treatment on day 0.

Supplementary Figure 6

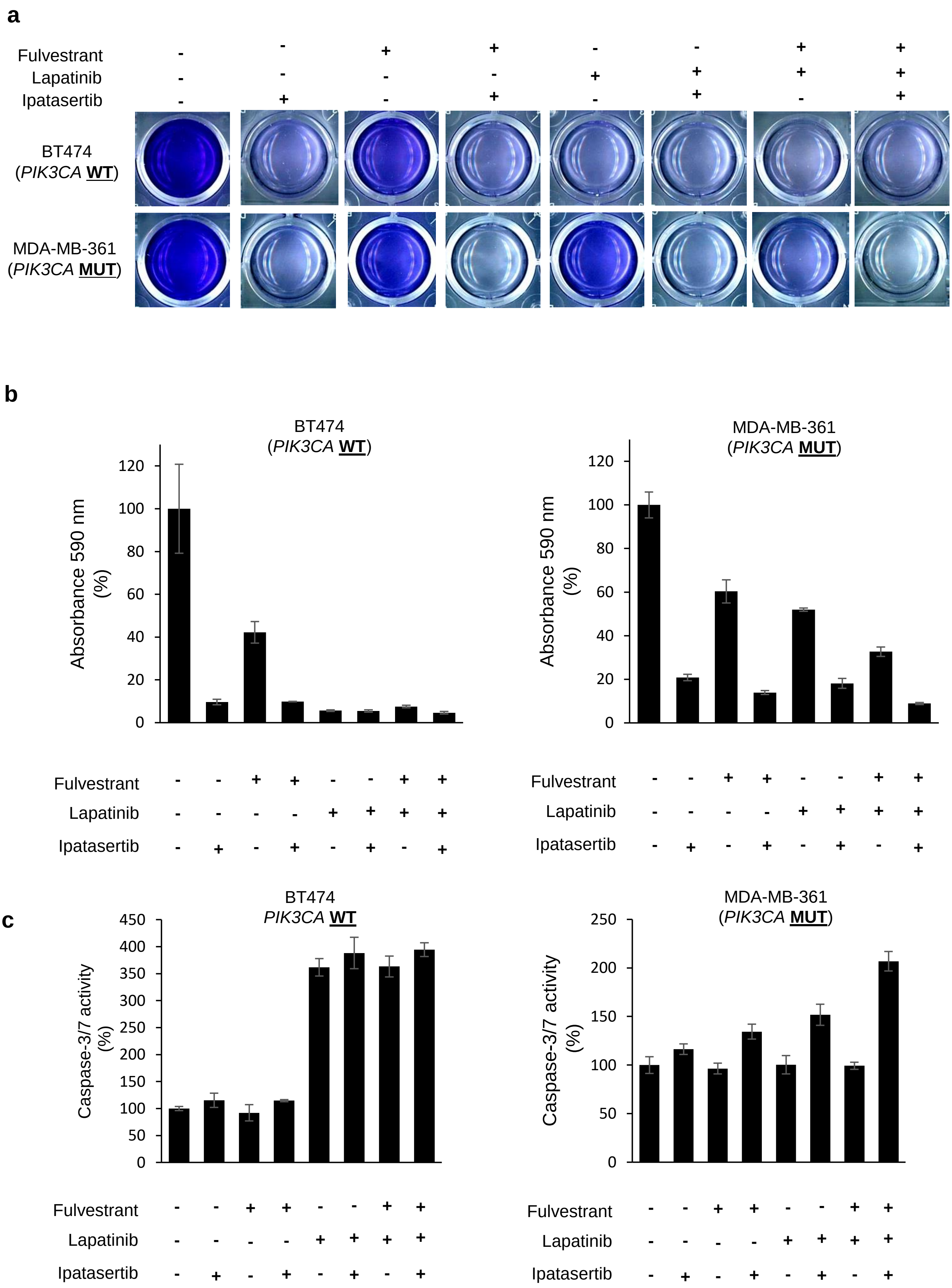

Supplementary Figure 6. Ipatasertib potently enhances antiproliferative activity of fulvestrant and lapatinib combination in a PIK3CA-mutant HER2+/ER+ breast cancer cell line (related to main figure 4c, 4d and 4e).

(a) Images of crystal violet staining in BT474 or MDA-MB-361 cells treated with fulvestrant, lapatinib, and ipatasertib. The cells were treated with alone or combination of the fulvestrant (100 nM), lapatinib (100 nM), and ipatasertib (1000 nM). Cells were collected 8 days after drug treatment for crystal violet staining. Plus (+) and minus (-) indicate the presence or absence of treatment. (b) Quantified data from Supplementary Fig. 6a. Crystal violet absorbance was measured with a microplate reader. The y axis indicates the amount of normalized protein, calculated based on absorbance compared with a DMSO control. The data represent mean ± SD (n = 3). (c) Effect of combined fulvestrant, lapatinib, and ipatasertib treatment on the apoptosis of BT474 (left) and MDA-MB-361 (right) cells. The cells were treated with alone or combination of DMSO, fulvestrant (100 nM), lapatinib (100 nM), and ipatasertib (1000 nM) for 24 h (mean ± SD [n = 3]). Relative caspase-3/7 activities were calculated based on luminescence compared with the DMSO control. The bars represent mean ± SD (n = 3).

Supplementary Figure 7

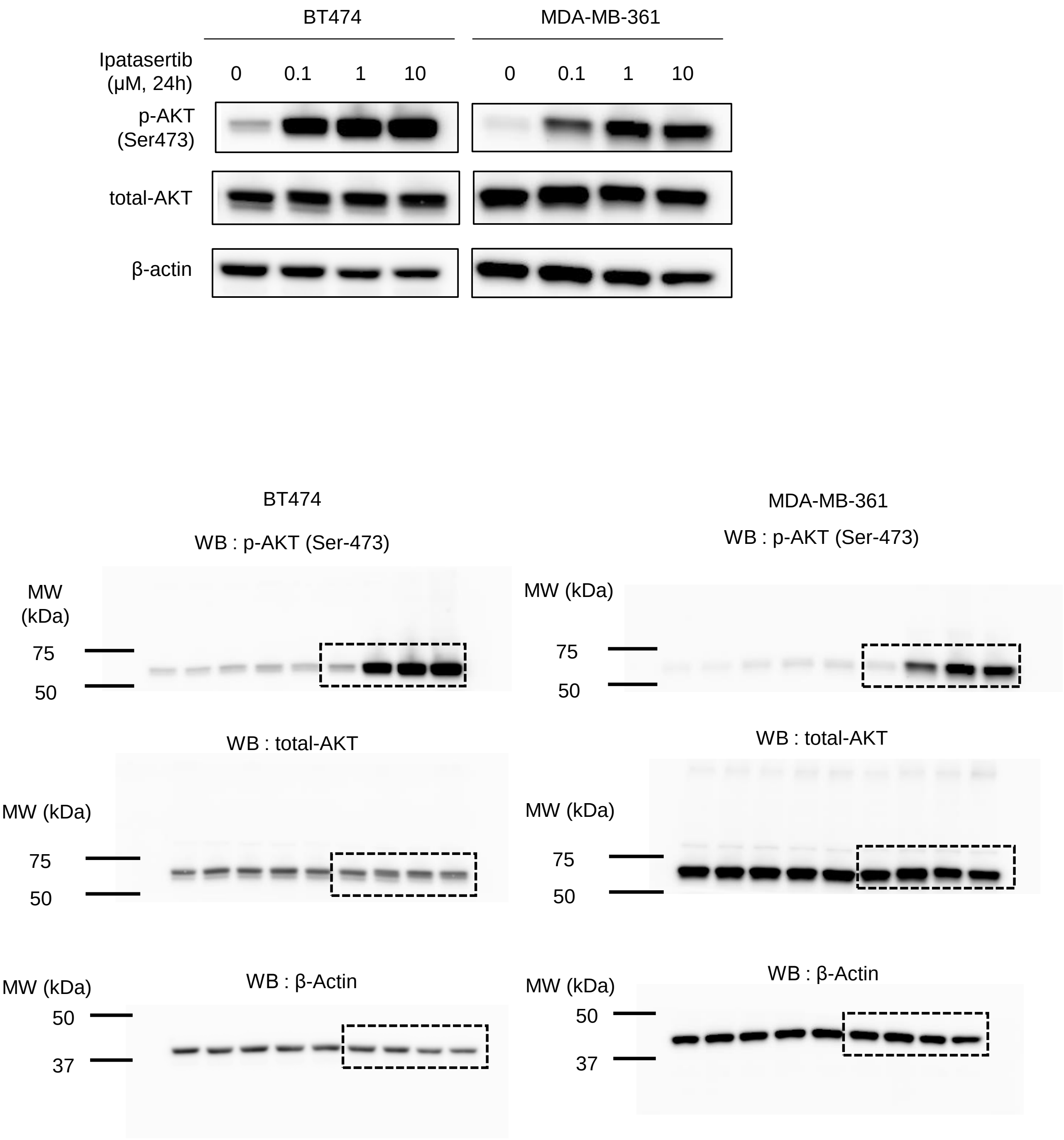

Supplementary Figure 7. Immunoblotting of pAKT at Ser473 in single-agent treatment with Ipatasertib (related to main figure 5).

Immunoblotting of pAKT at Ser473 in the Ipatasertib-treated BT474 (left) and MDA-MB-361 (right) cells. The cells were treated with ipatasertib at 0, 0.1, 1, and 10 μM for 24 h. pAKT at Ser473 was used as a pharmacodynamic marker to confirm target engagement of ipatasertib. AKT and β actin were used for a loading control.

Supplementary Figure 8

BT474 (*PIK3CA* **WT**)

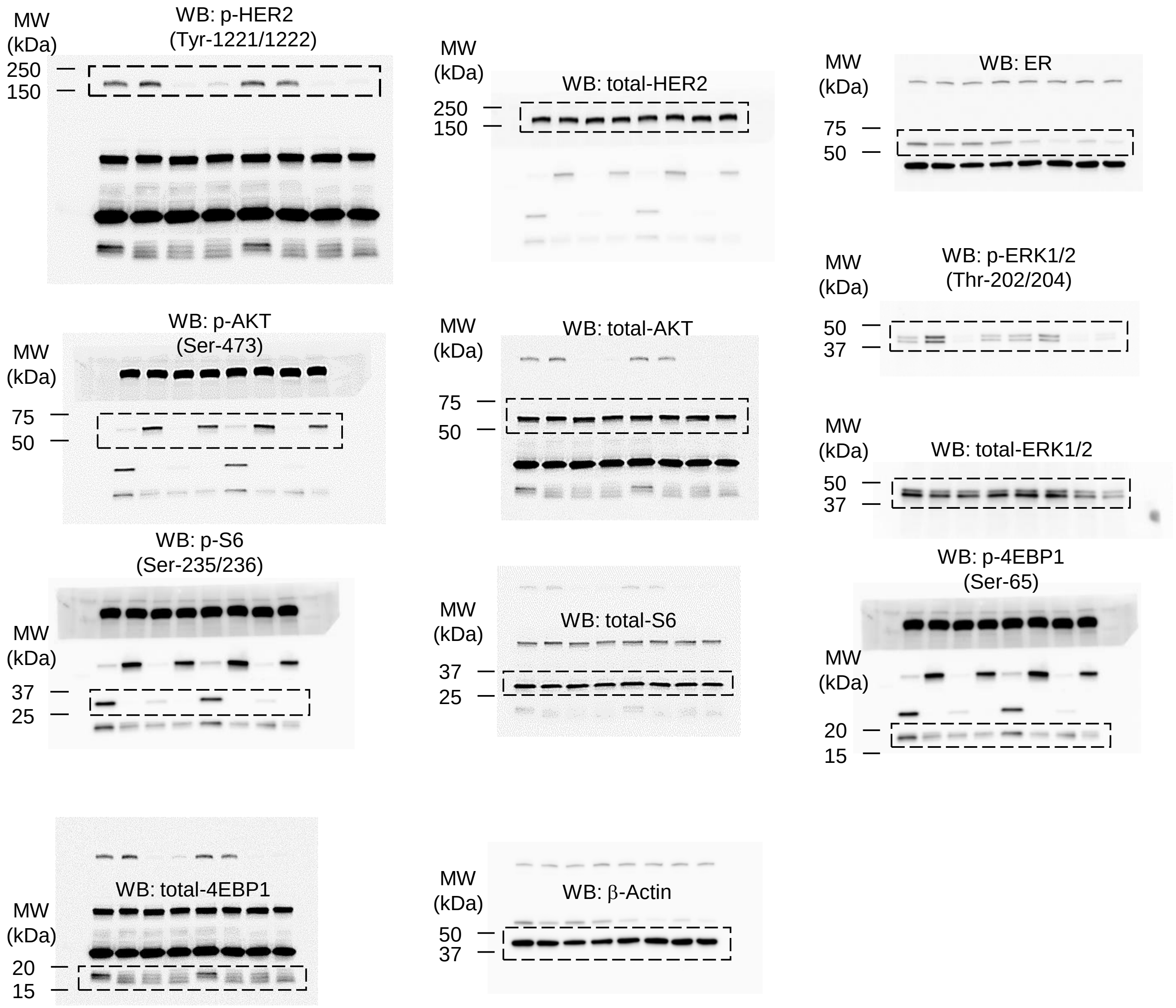

Supplementary Figure 8. Original images of immunoblots for main figure. 5a

Related to main figure 5a, BT474.

Supplementary Figure 9

MDA-MB-361 (*PIK3CA* **MUT**)

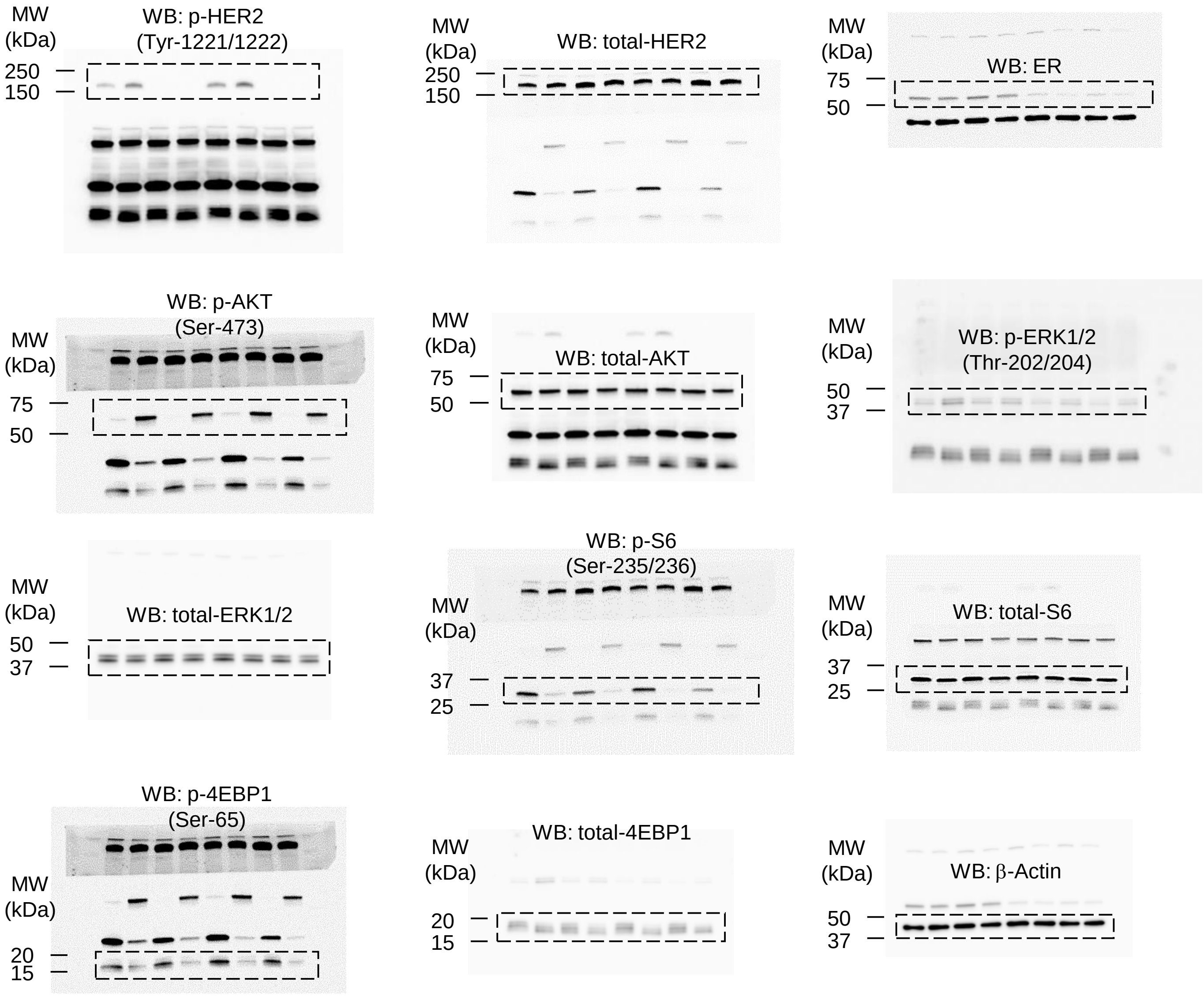

Supplementary Figure 9. Original images of immunoblots for main figure. 5a

Related to main figure 5a, MDA-MB-361.

Supplementary Figure 10

m^7GTP Pull down

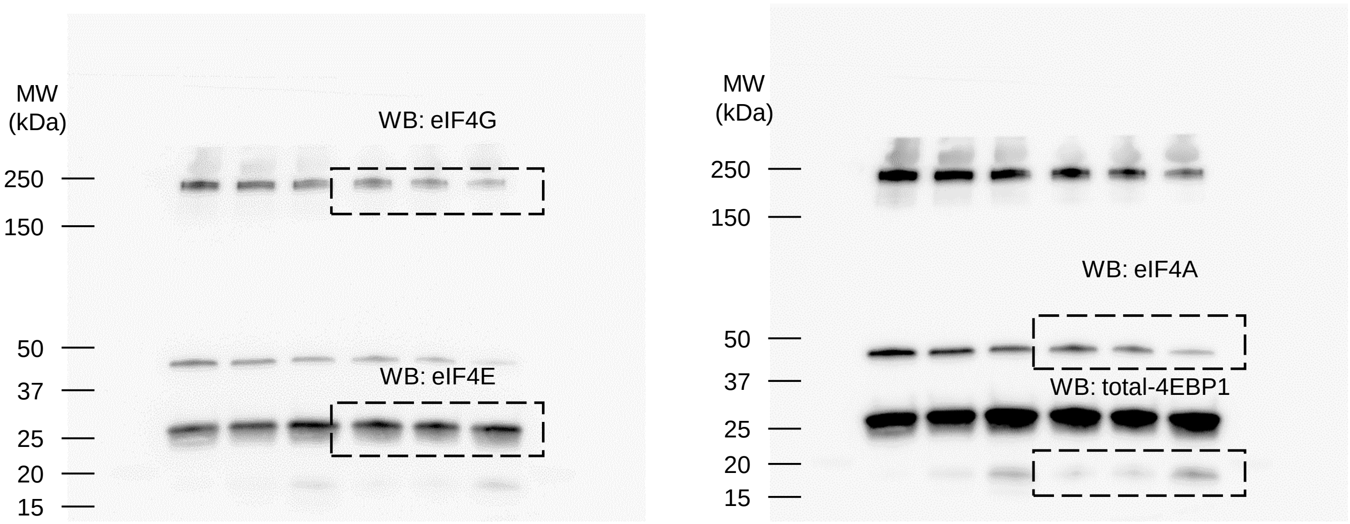

Input (cell lysate)

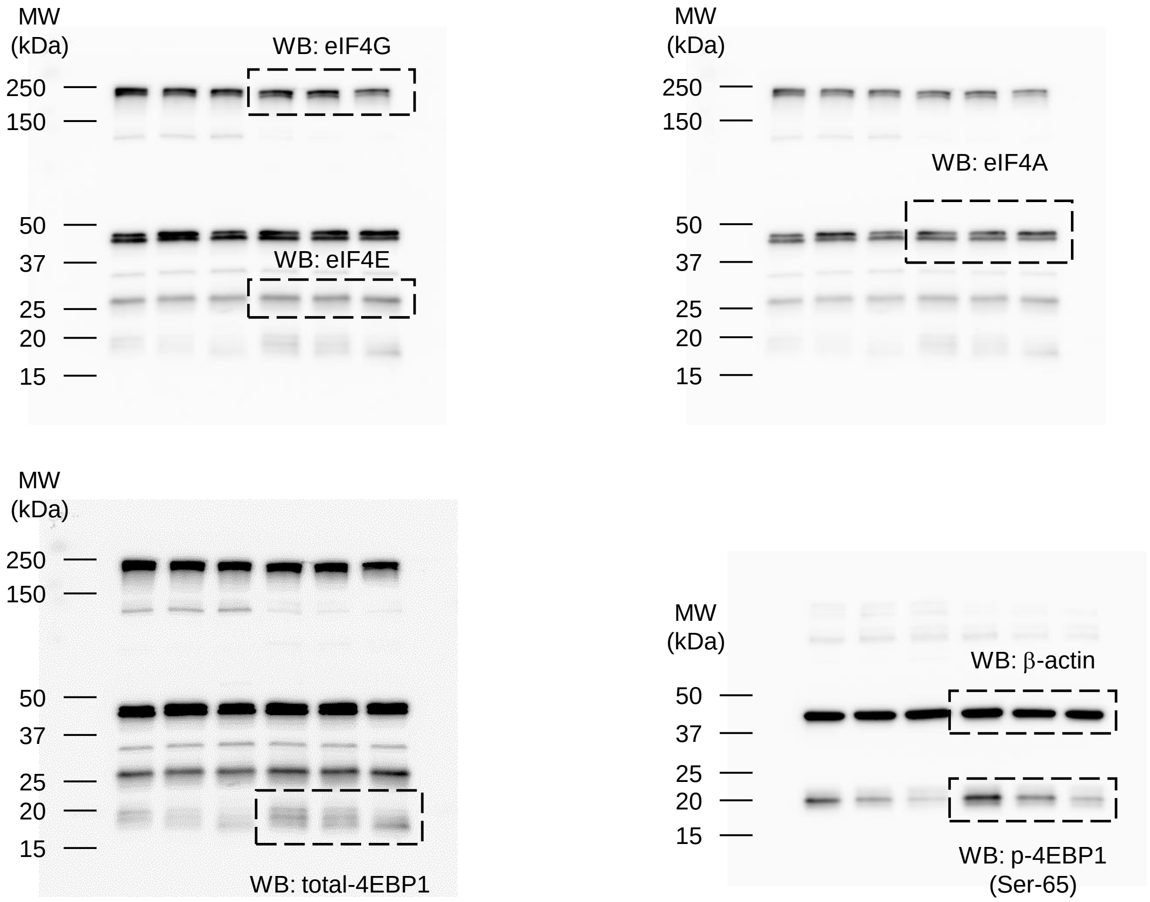

Supplementary Figure 10. Original images of immunoblots for main figure. 5e

Related to main figure 5e, m^7GTP pulldown and input (cell lysate).

Supplementary Figure 11

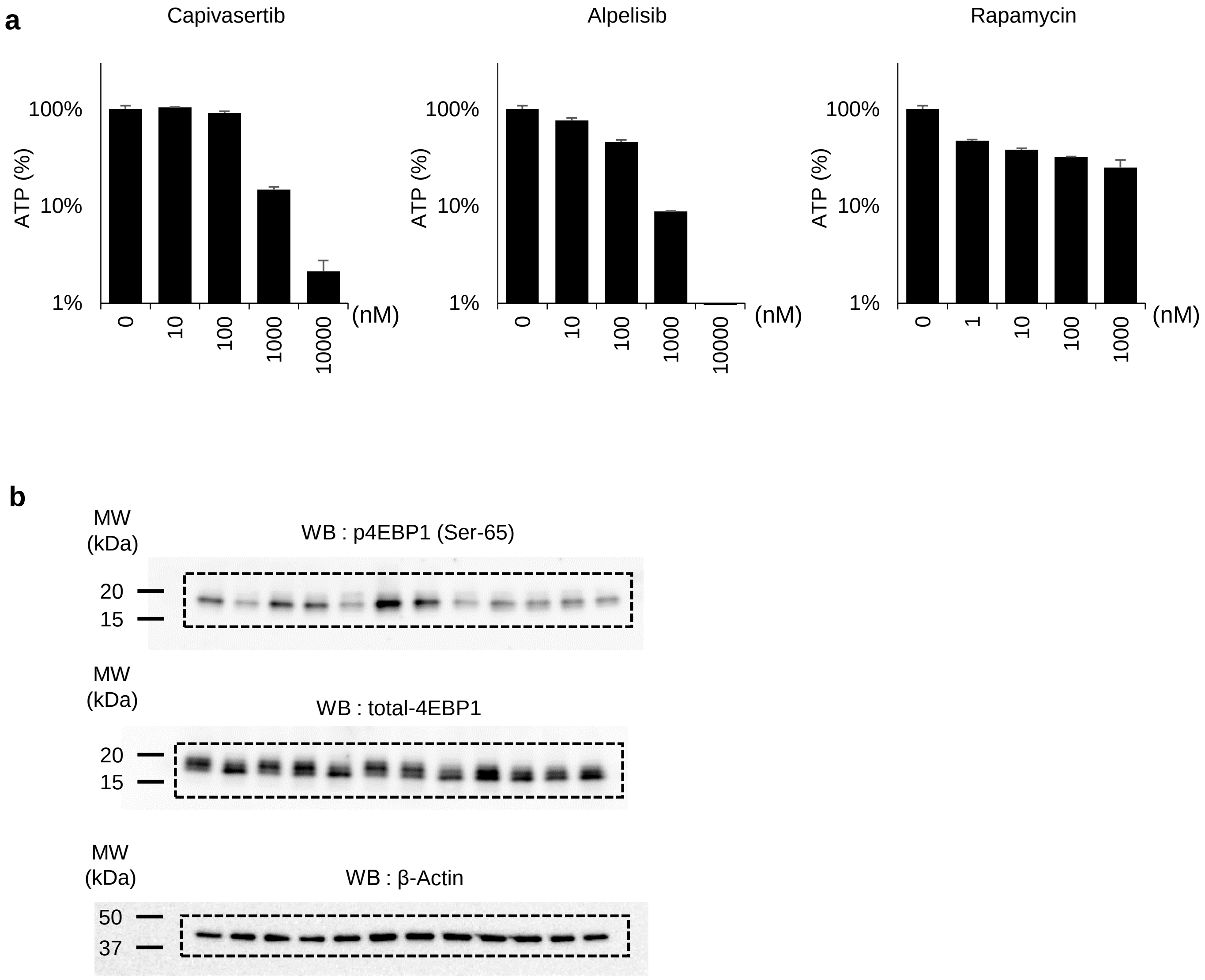

Supplementary Figure 11. Antiproliferative activity of PI3K pathway inhibitors (related to main figure 7).

(a) Antiproliferative activity of single treatment with Capivasertib, Alpelisib, and Rapamycin in MDA-MB-361 (*PIK3CA*-mutant) cells. Capivasertib, alpelisib, and rapamycin are AKT, PI3K, and mTOR inhibitors, respectively. The cells were treated for 8 days. Relative ATP amounts were calculated using a chemiluminescence assay and compared with the chemiluminescence value of DMSO treatment on day 8 (mean ± SD [n = 3]). (b) Original images of immunoblots for main figure. 7d

Table S1 Patient-level clinical data and treatment data for this study cohort extracted from Razavi et al. 2018 Cancer Cell

Patient_ID	Sample_ID	Sample_Type	Sample_Site	Metastatic_Dz	Gender	Invasive_Carcinoma Dx_Age	Receptor_Status Patient	ER_Status Patient	ER_Status Primary	ER_Status Sample	HER2_Status Patient	HER2_Status Primary
P-0000041	P-0000041-T01-IM3	Metastasis	Breast	Yes	Female	43	HR+/HER2+	Positive	Positive	Positive	Positive	Positive
P-0000058	P-0000058-T01-IM3	Metastasis	Liver	Yes	Female	49	HR+/HER2+	Positive	Positive	Positive	Positive	Positive
P-0000077	P-0000077-T01-IM3	Metastasis	Lymph Node	Yes	Female	60	HR+/HER2+	Positive	Positive	Positive	Positive	Positive
P-0000129	P-0000129-T01-IM3	Primary	Post-Treatment Primary	Yes	Female	46	HR+/HER2+	Positive	Positive	Positive	Positive	Negative
P-0000131	P-0000131-T01-IM3	Metastasis	Lymph Node	Yes	Female	61	HR+/HER2+	Positive	Positive	Positive	Positive	Positive
P-0000158	P-0000158-T01-IM3	Metastasis	Bone	Yes	Female	36	HR+/HER2+	Positive	Positive	Positive	Positive	Positive
P-0000215	P-0000215-T01-IM3	Metastasis	Liver	Yes	Female	47	HR+/HER2+	Positive	Positive	Positive	Positive	Positive
P-0000244	P-0000244-T01-IM3	Metastasis	Brain	Yes	Female	45	HR-/HER2+	Negative	Negative	Negative	Positive	Positive
P-0000295	P-0000295-T01-IM3	Metastasis	Liver	Yes	Female	50	HR+/HER2+	Positive	Positive	Negative	Positive	Positive
P-0000296	P-0000296-T01-IM3	Primary	Post-Neo Primary	Yes	Female	40	HR+/HER2+	Positive	Positive	Positive	Positive	Positive
P-0000358	P-0000358-T01-IM3	Primary	Treatment Naive Primary	Yes	Female	55	HR+/HER2+	Positive	Positive	Positive	Positive	Positive
P-0000411	P-0000411-T01-IM3	Metastasis	Soft Tissue	Yes	Female	59	HR+/HER2+	Positive	Positive	Negative	Positive	Positive
P-0000440	P-0000440-T01-IM3	Metastasis	Chest Wall	Yes	Female	53	HR-/HER2+	Negative	Negative	Negative	Positive	Negative
P-0000470	P-0000470-T02-IM5	Primary	Post-Treatment Primary	Yes	Female	66	HR+/HER2+	Positive	Positive	Positive	Positive	Positive
P-0000478	P-0000478-T01-IM3	Metastasis	Lung	Yes	Female	65	HR-/HER2+	Negative	Negative	Negative	Positive	Positive
P-0000483	P-0000483-T01-IM3	Metastasis	Breast	Yes	Female	50	HR+/HER2+	Positive	Positive	Positive	Positive	Negative
P-0000547	P-0000547-T01-IM3	Metastasis	Bone	Yes	Female	46	HR+/HER2+	Positive	Positive	Positive	Positive	Negative
P-0000550	P-0000550-T05-IM3	Metastasis	Lymph Node	Yes	Female	51	HR+/HER2+	Positive	Positive	Negative	Positive	Positive
P-0000606	P-0000606-T01-IM3	Metastasis	Lung	Yes	Female	33	HR+/HER2+	Positive	Positive	Positive	Positive	Positive
P-0000613	P-0000613-T01-IM3	Metastasis	Pleura	Yes	Female	36	HR+/HER2+	Positive	Positive	Negative	Positive	Positive
P-0000638	P-0000638-T01-IM3	Metastasis	Pericardium	Yes	Female	33	HR-/HER2+	Negative	Negative	Negative	Positive	Positive
P-0000656	P-0000656-T01-IM3	Metastasis	Liver	Yes	Female	50	HR+/HER2+	Positive	Unk/ND	Positive	Positive	Unk/ND
P-0000806	P-0000806-T01-IM3	Metastasis	Liver	Yes	Female	56	HR+/HER2+	Positive	Unk/ND	Positive	Positive	Unk/ND
P-0000969	P-0000969-T01-IM3	Metastasis	cal Recurrence/Lymph Nc	Yes	Female	31	HR+/HER2+	Positive	Positive	Positive	Positive	Negative
P-0001152	P-0001152-T01-IM3	Metastasis	Brain	Yes	Female	48	HR-/HER2+	Negative	Negative	Negative	Positive	Positive
P-0001156	P-0001156-T01-IM3	Metastasis	Lymph Node	Yes	Female	51	HR+/HER2+	Positive	Positive	Positive	Positive	Positive
P-0001218	P-0001218-T01-IM3	Metastasis	Liver	Yes	Female	45	HR+/HER2+	Positive	Positive	Positive	Positive	Negative
P-0001226	P-0001226-T01-IM3	Metastasis	Liver	Yes	Female	50	HR+/HER2+	Positive	Positive	Negative	Positive	Positive
P-0001335	P-0001335-T01-IM3	Primary	Treatment Naive Primary	Yes	Female	73	HR+/HER2+	Positive	Positive	Positive	Positive	Positive
P-0001338	P-0001338-T01-IM3	Metastasis	Soft Tissue	Yes	Female	40	HR+/HER2+	Positive	Positive	Positive	Positive	Positive
P-0001383	P-0001383-T01-IM3	Metastasis	Liver	Yes	Female	44	HR+/HER2+	Positive	Positive	Positive	Positive	Positive
P-0001415	P-0001415-T01-IM3	Primary	Treatment Naive Primary	Yes	Female	53	HR+/HER2+	Positive	Positive	Positive	Positive	Positive
P-0001483	P-0001483-T01-IM3	Primary	Treatment Naive Primary	Yes	Female	51	HR+/HER2+	Positive	Positive	Positive	Positive	Positive
P-0001488	P-0001488-T01-IM3	Metastasis	Lymph Node	Yes	Female	33	HR-/HER2+	Negative	Negative	Negative	Positive	Positive
P-0001504	P-0001504-T02-IM5	Metastasis	Chest Wall	Yes	Female	50	HR+/HER2+	Positive	Positive	Positive	Positive	Unk/ND
P-0001558	P-0001558-T01-IM3	Metastasis	Bone	Yes	Female	48	HR+/HER2+	Positive	Positive	Positive	Positive	Positive
P-0001559	P-0001559-T01-IM3	Metastasis	Lung	Yes	Female	37	HR+/HER2+	Positive	Positive	Positive	Positive	Positive
P-0001568	P-0001568-T01-IM3	Metastasis	Chest Wall	Yes	Female	47	HR+/HER2+	Positive	Positive	Negative	Positive	Positive
P-0001623	P-0001623-T01-IM3	Metastasis	Pleura	Yes	Female	46	HR+/HER2+	Positive	Positive	Positive	Positive	Positive
P-0001627	P-0001627-T01-IM3	Primary	Post-Treatment Primary	Yes	Female	58	HR-/HER2+	Negative	Negative	Negative	Positive	Positive
P-0001637	P-0001637-T01-IM3	Metastasis	Bone	Yes	Female	52	HR-/HER2+	Negative	Negative	Negative	Positive	Positive
P-0001715	P-0001715-T01-IM3	Metastasis	Chest Wall	Yes	Female	59	HR+/HER2+	Positive	Positive	Positive	Positive	Positive
P-0001718	P-0001718-T01-IM3	Primary	Treatment Naive Primary	Yes	Female	42	HR+/HER2+	Positive	Positive	Positive	Positive	Positive
P-0001757	P-0001757-T01-IM3	Metastasis	Liver	Yes	Female	65	HR-/HER2+	Negative	Negative	Negative	Positive	Positive
P-0001762	P-0001762-T01-IM3	Primary	Treatment Naive Primary	Yes	Female	36	HR+/HER2+	Positive	Positive	Positive	Positive	Positive
P-0001777	P-0001777-T01-IM3	Metastasis	Bone	Yes	Female	52	HR+/HER2+	Positive	Positive	Positive	Positive	Positive
P-0001785	P-0001785-T02-IM3	Metastasis	Lung	Yes	Female	40	HR+/HER2+	Positive	Positive	Negative	Positive	Negative (Outside Positive)
P-0001797	P-0001797-T01-IM3	Metastasis	Bone	Yes	Female	54	HR+/HER2+	Positive	Positive	Positive	Positive	Positive
P-0001818	P-0001818-T01-IM3	Primary	Treatment Naive Primary	Yes	Female	68	HR+/HER2+	Positive	Positive	Positive	Positive	Positive
P-0001860	P-0001860-T01-IM3	Metastasis	Liver	Yes	Female	58	HR-/HER2+	Negative	Negative	Negative	Positive	Positive
P-0001862	P-0001862-T01-IM3	Metastasis	Soft Tissue	Yes	Female	34	HR+/HER2+	Positive	Positive	Positive	Positive	Negative
P-0001883	P-0001883-T01-IM3	Metastasis	Brain	Yes	Female	43	HR-/HER2+	Negative	Negative	Negative	Positive	Positive
P-0001915	P-0001915-T01-IM3	Metastasis	Brain	Yes	Female	48	HR+/HER2+	Positive	Positive	Positive	Positive	Positive
P-0001939	P-0001939-T02-IM5	Metastasis	Lung	Yes	Female	38	HR+/HER2+	Positive	Positive	Positive	Positive	Negative
P-0001962	P-0001962-T01-IM3	Metastasis	Pleura	Yes	Female	41	HR+/HER2+	Positive	Positive	Positive	Positive	Positive
P-0002023	P-0002023-T01-IM3	Primary	Post-Neo Primary	Yes	Female	29	HR+/HER2+	Positive	Positive	Positive	Positive	Positive
P-0002036	P-0002036-T01-IM3	Metastasis	Brain	Yes	Female	33	HR-/HER2+	Negative	Negative	Negative	Positive	Positive
P-0002040	P-0002040-T01-IM3	Metastasis	Lung	Yes	Female	47	HR-/HER2+	Negative	Negative	Negative	Positive	Positive
P-0002177	P-0002177-T01-IM3	Metastasis	Liver	Yes	Female	60	HR+/HER2+	Positive	Negative	Positive	Positive	Positive
P-0002228	P-0002228-T01-IM3	Metastasis	Lymph Node	Yes	Female	62	HR+/HER2+	Positive	Positive	Positive	Positive	Negative
P-0002362	P-0002362-T01-IM3	Primary	Post-Neo Primary	Yes	Female	28	HR+/HER2+	Positive	Positive	Positive	Positive	Positive
P-0002387	P-0002387-T01-IM3	Metastasis	Chest Wall	Yes	Female	38	HR+/HER2+	Positive	Positive	Positive	Positive	Positive
P-0002390	P-0002390-T01-IM3	Metastasis	Soft Tissue	Yes	Female	27	HR+/HER2+	Positive	Positive	Positive	Positive	Positive
P-0002435	P-0002435-T01-IM3	Metastasis	Lung	Yes	Female	61	HR-/HER2+	Negative	Negative	Negative	Positive	Positive
P-0002447	P-0002447-T01-IM3	Metastasis	Brain	Yes	Female	43	HR+/HER2+	Positive	Positive	Positive	Positive	Positive
P-0002478	P-0002478-T01-IM3	Metastasis	Ovary	Yes	Female	52	HR+/HER2+	Positive	Positive	Positive	Positive	Positive
P-0002514	P-0002514-T01-IM3	Metastasis	Chest Wall	Yes	Female	46	HR+/HER2+	Positive	Positive	Positive	Positive	Positive
P-0002530	P-0002530-T01-IM3	Metastasis	Liver	Yes	Female	33	HR+/HER2+	Positive	Positive	Positive	Positive	Negative
P-0002538	P-0002538-T01-IM3	Metastasis	Chest Wall	Yes	Female	64	HR+/HER2+	Positive	Positive	Positive	Positive	Positive
P-0002567	P-0002567-T01-IM3	Metastasis	Liver	Yes	Female	46	HR+/HER2+	Positive	Positive	Negative	Positive	Positive
P-0002574	P-0002574-T01-IM3	Metastasis	Brain	Yes	Male	59	HR+/HER2+	Positive	Positive	Positive	Positive	Negative
P-0002626	P-0002626-T01-IM3	Metastasis	Lung	Yes	Female	56	HR+/HER2+	Positive	Positive	Positive	Positive	Positive
P-0002767	P-0002767-T01-IM3	Metastasis	Liver	Yes	Female	40	HR+/HER2+	Positive	Unk/ND	Positive	Positive	Unk/ND
P-0002789	P-0002789-T01-IM3	Metastasis	Chest Wall	Yes	Female	41	HR-/HER2+	Negative	Negative	Unk/ND	Positive	Positive
P-0002841	P-0002841-T01-IM3	Metastasis	Lymph Node	Yes	Female	28	HR+/HER2+	Positive	Positive	Positive	Positive	Positive
P-0002848	P-0002848-T01-IM3	Metastasis	Skin	Yes	Female	57	HR-/HER2+	Negative	Negative	Unk/ND	Positive	Positive
P-0002858	P-0002858-T01-IM3	Metastasis	Lymph Node	Yes	Female	55	HR+/HER2+	Positive	Positive	Negative	Positive	Unk/ND
P-0002995	P-0002995-T01-IM3	Metastasis	Lymph Node	Yes	Female	46	HR+/HER2+	Positive	Positive	Positive	Positive	Positive
P-0002997	P-0002997-T01-IM3	Metastasis	Liver	Yes	Female	46	HR+/HER2+	Positive	Positive	Positive	Positive	Equivocal
P-0003057	P-0003057-T01-IM5	Metastasis	Chest Wall	Yes	Female	47	HR+/HER2+	Positive	Positive	Positive	Positive	Negative
P-0003198	P-0003198-T02-IM5	Metastasis	Chest Wall	Yes	Female	37	HR-/HER2+	Negative	Negative	Negative	Positive	Positive
P-0003286	P-0003286-T01-IM5	Metastasis	Skin	Yes	Female	73	HR+/HER2+	Positive	Positive	Positive	Positive	Positive
P-0003353	P-0003353-T01-IM5	Metastasis	Brain	Yes	Female	52	HR-/HER2+	Negative	Negative	Negative	Positive	Positive
P-0003509	P-0003509-T01-IM5	Primary	Treatment Naive Primary	Yes	Female	50	HR+/HER2+	Positive	Positive	Positive	Positive	Positive
P-0003570	P-0003570-T01-IM5	Metastasis	Brain	Yes	Female	39	HR+/HER2+	Positive	Unk/ND	Positive	Positive	Unk/ND
P-0003624	P-0003624-T01-IM5	Metastasis	Soft Tissue	Yes	Female	49	HR-/HER2+	Negative	Negative	Unk/ND	Positive	Positive
P-0003647	P-0003647-T01-IM5	Primary	Post-Treatment Primary	Yes	Female	43	HR+/HER2+	Positive	Positive	Positive	Positive	Positive
P-0003702	P-0003702-T02-IM5	Metastasis	Epidural Mass	Yes	Female	50	HR+/HER2+	Positive	Negative	Positive	Positive	Positive
P-0003761	P-0003761-T01-IM5	Metastasis	Liver	Yes	Female	32	HR+/HER2+	Positive	Positive	Positive	Positive	Positive
P-0003986	P-0003986-T01-IM5	Primary	Treatment Naive Primary	Yes	Female	53	HR+/HER2+	Positive	Positive	Positive	Positive	Positive
P-0004057	P-0004057-T01-IM5	Metastasis	Liver	Yes	Female	73	HR+/HER2+	Positive	Positive	Positive	Positive	Positive
P-0004104	P-0004104-T01-IM5	Metastasis	Soft Tissue	Yes	Female	32	HR+/HER2+	Positive	Positive	Positive	Positive	Positive
P-0004133	P-0004133-T01-IM5	Primary	Treatment Naive Primary	Yes	Female	48	HR-/HER2+	Negative	Negative	Negative	Positive	Positive
P-0004160	P-0004160-T01-IM5	Metastasis	Lymph Node	Yes	Female	46	HR-/HER2+	Negative	Negative	Negative	Positive	Positive
P-0004180	P-0004180-T01-IM5	Metastasis	Liver	Yes	Female	60	HR+/HER2+	Positive	Positive	Positive	Positive	Positive
P-0004210	P-0004210-T01-IM5	Primary	Treatment Naive Primary	Yes	Female	45	HR-/HER2+	Negative	Negative	Negative	Positive	Positive
P-0004265	P-0004265-T01-IM5	Metastasis	Liver	Yes	Female	52	HR+/HER2+	Positive	Positive	Positive	Positive	Negative
P-0004293	P-0004293-T01-IM5	Metastasis	Peritoneum	Yes	Female	69	HR+/HER2+	Positive	Positive	Positive	Positive	Positive
P-0004310	P-0004310-T01-IM5	Primary	Post-Neo Primary	Yes	Female	35	HR+/HER2+	Positive	Positive	Positive	Positive	Positive
P-0004317	P-0004317-T01-IM5	Metastasis	Lymph Node	Yes	Female	55	HR+/HER2+	Positive	Positive	Positive	Positive	Positive
P-0004381	P-0004381-T01-IM5	Primary	Treatment Naive Primary	Yes	Female	41	HR+/HER2+	Positive	Positive	Positive	Positive	Unk/ND
P-0004455	P-0004455-T01-IM5	Primary	Treatment Naive Primary	Yes	Female	59	HR+/HER2+	Positive	Positive	Positive	Positive	Positive
P-0004459	P-0004459-T01-IM5	Metastasis	Liver	Yes	Female	45	HR+/HER2+	Positive	Positive	Positive	Positive	Positive
P-0004466	P-0004466-T01-IM5	Metastasis	Chest Wall	Yes	Female	36	HR-/HER2+	Negative	Negative	Negative	Positive	Positive
P-0004540	P-0004540-T01-IM5	Primary	Post-Treatment Primary	Yes	Female	56	HR+/HER2+	Positive	Positive	Positive	Positive	Positive
P-0004576	P-0004576-T01-IM5	Primary	Treatment Naive Primary	Yes	Female	38	HR+/HER2+	Positive	Positive	Positive	Positive	Positive
P-0004589	P-0004589-T01-IM5	Metastasis	Pleura	Yes	Female	56	HR+/HER2+	Positive	Positive	Positive	Positive	Positive

HER2_Status Sample	Treatment_Regimen	Anti-HER2-Treatmen Start_Time	Last_Contact_Time	Death_Time	OS_Day	OS_Month	OS_Event	PIK3CA Mutation	PIK3R1 Mutation	AKT1 Mutation	PTEN Loss_or_Mutation	PI3K_Pathway Mutation_Status
Negative	Fulvestrant-Trastuzumab	18363	19364	19364	1001	32.9	1	1	0	0	0	1
Positive	THP	18067	19297		1230	40.4	0	0	0	0	0	0
Positive	THP->HP	26800	27809		1009	33.2	0	0	0	0	0	0
Positive	THP->HP	17826	18029	18029	203	6.7	1	0	0	0	1	1
Positive	THP	23983	25038		1055	34.7	0	0	0	0	1	1
Positive	Tamoxifen-Trastuzumab	14598	15626		1028	33.8	0	0	0	0	0	0
Positive	Tamoxifen-Trastuzumab	17163	19498		2335	76.8	0	1	0	0	0	1
Positive	TH->Trastuzumab	16507	18939		2432	80.0	0	0	0	0	0	0
Positive	Carboplatin-Docetaxel-Trastuzumab	18446	20698		2252	74.0	0	1	0	0	0	1
Positive	THP->HP	15118	15334		216	7.1	0	0	0	0	0	0
Positive	Vinorelbine-Trastuzumab	21964	23838		1874	61.6	0	0	0	0	0	0
Positive	THP	23178	24120		942	31.0	0	0	1	0	0	1
Positive	Carboplatin-Docetaxel-Trastuzumab	21101	24507	24507	3406	112.0	1	1	0	0	0	1
Positive	THP->HP	24194	25225		1031	33.9	0	1	0	0	0	1
Positive	TH->Trastuzumab	23852	25856	25856	2004	65.9	1	0	0	0	0	0
Positive	Capecitabine-Trastuzumab	21091	21973		882	29.0	0	0	0	0	0	0
Negative	Nab paclitaxel-Trastuzumab	20946	23154	23154	2208	72.6	1	1	0	0	0	1
Positive	Capecitabine-Trastuzumab	22289	24152		1863	61.2	0	0	0	0	0	0
Positive	THP->Tamoxifen-HP	12111	13150		1039	34.2	0	1	0	0	0	1
Positive	Capecitabine-Trastuzumab	14818	17236	17236	2418	79.5	1	1	0	0	0	1
Positive	THP->HP	12633	13284	13284	651	21.4	1	0	0	0	0	0
Positive	TH	18370	20396	20396	2026	66.6	1	0	0	0	0	0
Positive	Capecitabine-Lapatinib	21404	22157		753	24.8	0	0	0	0	0	0
Positive	Tamoxifen-Trastuzumab	11898	12821		923	30.3	0	0	0	0	0	0
Positive	Trastuzumab	18768	19644		876	28.8	0	0	0	0	0	0
Positive	THP	19638	20088	20088	450	14.8	1	1	0	0	0	1
Negative	THP	18320	18491	18491	171	5.6	1	1	0	0	0	1
Positive	TH	21040	22214	22214	1174	38.6	1	1	0	0	0	1
Positive	THP->HP	26549	26815		266	8.7	0	1	0	0	0	1
Positive	THP->Tamoxifen-HP	15065	15888		823	27.1	0	1	0	0	0	1
Positive	THP->Letrozole-HP	16256	17253		997	32.8	0	1	0	0	0	1
Positive	THP->HP	23844	24180		336	11.0	0	0	0	0	0	0
Positive	THP->HP	19798	20617		819	26.9	0	0	0	0	0	0
Positive	Carboplatin-Docetaxel-Trastuzumab	13604	17828		4224	138.9	0	1	0	0	0	1
Positive	THP->HP	23445	24115		670	22.0	0	0	0	0	0	0
Positive	Exemestane-Trastuzumab	19624	21289		1665	54.7	0	0	0	0	0	0
Positive	THP	13480	14958		1478	48.6	0	1	0	0	0	1
Positive	Vinorelbine-Trastuzumab	18579	22176		3597	118.3	0	1	0	0	0	1
Positive	Letrozole-Trastuzumab	18052	18912		860	28.3	0	1	0	0	0	1
Positive	THP	21083	21959		876	28.8	0	1	0	0	0	1
Positive	THP->HP	19274	20171		897	29.5	0	0	1	0	0	1
Positive	Trastuzumab	23881	26403		2522	82.9	0	0	0	0	0	0
Unk/ND	Letrozole-Trastuzumab	15488	17960		2472	81.3	0	0	0	0	1	1
Positive	Vinorelbine-Trastuzumab	23576	24944	24944	1368	45.0	1	0	0	0	0	0
Positive	THP->HP	13244	14018		774	25.4	0	1	0	0	0	1
Equivocal	THP	19682	20034	20034	352	11.6	1	0	0	0	0	0
Negative	THP	15477	16338		861	28.3	0	0	0	0	0	0
Negative	Tamoxifen-Trastuzumab	22086	23713	23713	1559	51.3	1	1	0	0	0	1
Positive	TH	24857	26539		1682	55.3	0	0	0	0	0	0
Positive	THP->HP	22144	23133	23133	989	32.5	1	0	0	0	0	0
Positive	Anastrozole-Trastuzumab	13224	14136		912	30.0	0	0	0	0	0	0
Positive	THP->HP	17931	18766		835	27.5	0	1	0	0	0	1
Positive	Vinorelbine-Trastuzumab	18598	20228		1630	53.6	0	0	0	0	0	0
Positive	Palbociclib-Fulvestrant-Trastuzumab	17557	17898		341	11.2	0	1	0	0	0	1
Positive	Trastuzumab	19868	21777		1909	62.8	0	0	0	0	0	0
Positive	TDM1	11072	11993		921	30.3	0	0	0	0	0	0
Positive	THP->HP	11992	12708	12708	716	23.5	1	0	0	0	0	0
Negative	emcitabine-Carboplatin-Trastuzuma	17872	18227	18227	355	11.7	1	1	0	0	0	1
Negative	Capecitabine-Trastuzumab	23406	25182		1776	58.4	0	0	0	0	0	0
Positive	Exemestane-Trastuzumab	25086	25997		498	16.4	0	0	0	0	0	0
Positive	Tamoxifen-Trastuzumab	10748	11712		964	31.7	0	0	0	0	0	0
Negative	Fulvestrant-Trastuzumab	16918	18233	18233	1315	43.2	1	1	0	0	0	1
Negative	TH	14222	17729	17729	3507	115.3	1	0	0	0	0	0
Negative	Lapatinib-Trastuzumab	24349	24713	24713	364	12.0	1	0	0	0	1	1
Positive	Tamoxifen-Trastuzumab	16500	17765		1265	41.6	0	0	0	0	0	0
Positive	THP->HP	20220	20986		766	25.2	0	0	0	0	1	1
Positive	Vinorelbine-Trastuzumab	18151	19986		1835	60.3	0	0	0	0	0	0
Positive	THP	17065	17634	17634	569	18.7	1	0	0	0	0	0
Positive	THP->Letrozole-HP	23370	24032		662	21.8	0	0	0	0	0	0
Positive	THP->HP	17560	17953		393	12.9	0	0	0	0	1	1
Positive	Exemestane-Trastuzumab	23515	25607		2092	68.8	0	0	0	0	0	0
Positive	THP->HP	20606	22058		1452	47.7	0	0	0	0	0	0
Positive	Lapatinib-Trastuzumab	15092	16707	16707	1615	53.1	1	0	0	0	0	0
Unk/ND	THP->HP	15467	16152	16152	685	22.5	1	1	0	0	0	1
Negative	THP->HP	11685	13610		1925	63.3	0	1	0	0	0	1
Unk/ND	Vinorelbine-Trastuzumab	21229	24796		3567	117.3	0	0	0	0	0	0
Positive	HP	27445	28284		839	27.6	0	1	0	1	0	1
Positive	Anastrozole-Trastuzumab	18533	20746		2213	72.8	0	0	0	0	0	0
Positive	TH->Trastuzumab	20029	23053		3024	99.4	0	0	0	0	0	0
Positive	Fulvestrant-Trastuzumab	18311	18585	18585	274	9.0	1	0	0	0	0	0
Positive	Carboplatin-Paclitaxel-Trastuzumab	14656	16819		2163	71.1	0	0	0	0	0	0
Positive	TH	26908	27650		742	24.4	0	1	0	0	0	1
Positive	Trastuzumab	19690	20804		1114	36.6	0	0	0	0	0	0
Positive	THP	18330	18878		548	18.0	0	0	0	0	0	0
Positive	Capecitabine-Trastuzumab	17286	18227		941	30.9	0	1	0	0	0	1
Unk/ND	THP	18962	20759		1797	59.1	0	1	0	0	0	1
Positive	Letrozole-Trastuzumab	15647	18310	18310	2663	87.6	1	0	0	0	0	0
Positive	Tamoxifen-Trastuzumab	18351	19172		821	27.0	0	0	0	0	0	0
Positive	THP->HP	11792	13206		1414	46.5	0	0	0	0	0	0
Positive	Anastrozole-HP	19544	20326		782	25.7	0	0	0	0	0	0
Negative	THP	26559	26616		57	1.9	0	0	0	0	0	0
Negative	Capecitabine-Trastuzumab	12367	14370	14370	2003	65.9	1	0	0	0	0	0
Positive	THP->HP	17486	18228		742	24.4	0	0	0	0	0	0
Positive	THP->HP	19301	20967		1666	54.8	0	0	0	0	0	0
Positive	THP->HP	23710	25348		1638	53.9	0	0	0	0	0	0
Positive	Trastuzumab	17629	17669	17669	40	1.3	1	0	0	0	0	0
Positive	THP	20306	20752	20752	446	14.7	1	0	0	0	0	0
Positive	Anastrozole-Trastuzumab	27353	28570	28570	1217	40.0	1	0	0	0	0	0
Positive	Letrozole-Trastuzumab	14735	15755		1020	33.5	0	1	0	0	0	1
Positive	TH->Trastuzumab	20128	24828		4700	154.5	0	0	0	0	0	0
Unk/ND	THP	19177	21123		1946	64.0	0	0	0	0	0	0
Positive	THP->HP	21587	22308		721	23.7	0	0	0	0	0	0
Positive	THP->HP	16507	18004		1497	49.2	0	0	0	0	0	0
Positive	THP	13622	14804		1182	38.9	0	1	0	0	0	1
Positive	Anastrozole-Trastuzumab	20603	22259		1656	54.4	0	0	0	0	0	0
Positive	THP->HP	14067	16208		2141	70.4	0	0	0	0	0	0
Positive	THP->HP	23038	23753		715	23.5	0	1	0	0	0	1

P-0004601	P-0004601-T01-IM5	Metastasis	Chest Wall	Yes	Female	53	HR-/HER2+	Negative	Negative	Negative	Positive	Positive
P-0004681	P-0004681-T01-IM5	Metastasis	Breast	Yes	Female	58	HR-/HER2+	Negative	Negative	Negative	Positive	Positive
P-0004702	P-0004702-T02-IM5	Metastasis	Liver	Yes	Female	38	HR+/HER2+	Positive	Positive	Positive	Positive	Negative
P-0004720	P-0004720-T01-IM5	Metastasis	Brain	Yes	Female	54	HR+/HER2+	Positive	Positive	Negative	Positive	Positive
P-0004841	P-0004841-T01-IM5	Primary	Treatment Naive Primary	Yes	Female	37	HR+/HER2+	Positive	Positive	Positive	Positive	Positive
P-0004912	P-0004912-T01-IM5	Metastasis	Brain	Yes	Female	42	HR+/HER2+	Positive	Positive	Unk/ND	Positive	Positive
P-0004918	P-0004918-T01-IM5	Primary	Treatment Naive Primary	Yes	Female	35	HR+/HER2+	Positive	Positive	Positive	Positive	Negative
P-0004921	P-0004921-T01-IM5	Primary	Treatment Naive Primary	Yes	Female	41	HR-/HER2+	Negative	Negative	Negative	Positive	Positive
P-0004965	P-0004965-T01-IM5	Primary	Treatment Naive Primary	Yes	Female	59	HR-/HER2+	Negative	Negative	Negative	Positive	Positive
P-0004975	P-0004975-T01-IM5	Metastasis	Liver	Yes	Female	54	HR+/HER2+	Positive	Positive	Positive	Positive	Positive
P-0005005	P-0005005-T01-IM5	Primary	Treatment Naive Primary	Yes	Female	53	HR+/HER2+	Positive	Positive	Positive	Positive	Positive
P-0005010	P-0005010-T01-IM5	Primary	Treatment Naive Primary	Yes	Female	33	HR-/HER2+	Negative	Negative	Negative	Positive	Positive
P-0005036	P-0005036-T01-IM5	Metastasis	Liver	Yes	Female	52	HR+/HER2+	Positive	Positive	Positive	Positive	Negative
P-0005079	P-0005079-T01-IM5	Primary	Post-Neo Primary	Yes	Female	41	HR-/HER2+	Negative	Negative	Negative	Positive	Positive
P-0005095	P-0005095-T01-IM5	Primary	Treatment Naive Primary	Yes	Female	53	HR+/HER2+	Positive	Positive	Positive	Positive	Positive
P-0005205	P-0005205-T01-IM5	Metastasis	Lung	Yes	Female	35	HR-/HER2+	Negative	Negative	Negative	Positive	Positive
P-0005221	P-0005221-T01-IM5	Metastasis	Chest Wall	Yes	Female	35	HR-/HER2+	Negative	Negative	Negative	Positive	Positive
P-0005268	P-0005268-T01-IM5	Metastasis	Bone	Yes	Female	46	HR+/HER2+	Positive	Unk/ND	Positive	Positive	Unk/ND
P-0005273	P-0005273-T01-IM5	Metastasis	Lymph Node	Yes	Female	53	HR-/HER2+	Negative	Negative	Negative	Positive	Positive
P-0005342	P-0005342-T01-IM5	Metastasis	Liver	Yes	Female	46	HR+/HER2+	Positive	Positive	Positive	Positive	Positive
P-0005351	P-0005351-T01-IM5	Primary	Treatment Naive Primary	Yes	Female	45	HR+/HER2+	Positive	Positive	Positive	Positive	Positive
P-0005419	P-0005419-T01-IM5	Metastasis	Lymph Node	Yes	Female	54	HR+/HER2+	Positive	Negative	Positive	Positive	Negative
P-0005602	P-0005602-T01-IM5	Metastasis	Soft Tissue	Yes	Female	39	HR+/HER2+	Positive	Positive	Positive	Positive	Positive
P-0005611	P-0005611-T01-IM5	Primary	Post-Treatment Primary	Yes	Female	50	HR-/HER2+	Negative	Negative	Negative	Positive	Positive
P-0005620	P-0005620-T02-IM5	Primary	Treatment Naive Primary	Yes	Female	44	HR-/HER2+	Negative	Negative	Negative	Positive	Positive
P-0005631	P-0005631-T01-IM5	Metastasis	Liver	Yes	Female	42	HR+/HER2+	Positive	Positive	Negative	Positive	Positive
P-0005691	P-0005691-T01-IM5	Metastasis	Bone	Yes	Female	56	HR+/HER2+	Positive	Positive	Positive	Positive	Positive
P-0005832	P-0005832-T01-IM5	Primary	Treatment Naive Primary	Yes	Female	38	HR-/HER2+	Negative	Negative	Negative	Positive	Positive
P-0005855	P-0005855-T01-IM5	Metastasis	Bone	Yes	Female	57	HR+/HER2+	Positive	Positive	Negative	Positive	Positive
P-0005859	P-0005859-T01-IM5	Primary	Treatment Naive Primary	Yes	Female	65	HR+/HER2+	Positive	Positive	Positive	Positive	Positive
P-0005940	P-0005940-T01-IM5	Metastasis	Bone	Yes	Female	33	HR+/HER2+	Positive	Positive	Positive	Positive	Positive
P-0006137	P-0006137-T01-IM5	Primary	Treatment Naive Primary	Yes	Female	49	HR+/HER2+	Positive	Positive	Positive	Positive	Positive
P-0006156	P-0006156-T01-IM5	Metastasis	Chest Wall	Yes	Female	58	HR+/HER2+	Positive	Positive	Positive	Positive	Positive
P-0006189	P-0006189-T01-IM5	Metastasis	Chest Wall	Yes	Female	37	HR+/HER2+	Positive	Positive	Positive	Positive	Positive
P-0006262	P-0006262-T01-IM5	Metastasis	Bone	Yes	Female	50	HR+/HER2+	Positive	Positive	Positive	Positive	Positive
P-0006335	P-0006335-T01-IM5	Metastasis	Lymph Node	Yes	Female	46	HR+/HER2+	Positive	Positive	Positive	Positive	Negative
P-0006455	P-0006455-T01-IM5	Metastasis	Liver	Yes	Female	55	HR-/HER2+	Negative	Negative	Negative	Positive	Positive
P-0006458	P-0006458-T01-IM5	Primary	Treatment Naive Primary	Yes	Female	52	HR-/HER2+	Negative	Negative	Negative	Positive	Positive
P-0006497	P-0006497-T01-IM5	Primary	Treatment Naive Primary	Yes	Female	75	HR+/HER2+	Positive	Positive	Positive	Positive	Positive
P-0006517	P-0006517-T01-IM5	Primary	Treatment Naive Primary	Yes	Female	31	HR+/HER2+	Positive	Positive	Positive	Positive	Positive
P-0006668	P-0006668-T01-IM5	Metastasis	Lung	Yes	Female	70	HR-/HER2+	Negative	Negative	Negative	Positive	Positive
P-0006676	P-0006676-T01-IM5	Metastasis	Liver	Yes	Female	23	HR+/HER2+	Positive	Positive	Positive	Positive	Positive
P-0006698	P-0006698-T03-IM5	Metastasis	Liver	Yes	Female	57	HR-/HER2+	Negative	Negative	Negative	Positive	Positive
P-0006857	P-0006857-T01-IM5	Metastasis	Brain	Yes	Female	30	HR-/HER2+	Negative	Negative	Negative	Positive	Positive
P-0006996	P-0006996-T01-IM5	Metastasis	Liver	Yes	Female	42	HR+/HER2+	Positive	Positive	Negative	Positive	Positive
P-0007045	P-0007045-T01-IM5	Metastasis	Lymph Node	Yes	Female	40	HR+/HER2+	Positive	Positive	Positive	Positive	Positive
P-0007089	P-0007089-T02-IM5	Metastasis	Lymph Node	Yes	Female	58	HR+/HER2+	Positive	Positive	Negative	Positive	Positive
P-0007093	P-0007093-T01-IM5	Metastasis	Liver	Yes	Female	58	HR-/HER2+	Negative	Negative	Unk/ND	Positive	Positive
P-0007127	P-0007127-T01-IM5	Metastasis	Lung	Yes	Female	69	HR-/HER2+	Negative	Negative	Unk/ND	Positive	Negative
P-0007146	P-0007146-T01-IM5	Metastasis	Liver	Yes	Female	37	HR+/HER2+	Positive	Positive	Positive	Positive	Positive
P-0007228	P-0007228-T01-IM5	Metastasis	Liver	Yes	Female	28	HR+/HER2+	Positive	Positive	Negative	Positive	Positive
P-0007335	P-0007335-T01-IM5	Metastasis	Liver	Yes	Female	69	HR+/HER2+	Positive	Negative	Positive	Positive	Positive
P-0007340	P-0007340-T01-IM5	Metastasis	Liver	Yes	Female	43	HR+/HER2+	Positive	Positive	Positive	Positive	Positive
P-0007349	P-0007349-T01-IM5	Primary	Treatment Naive Primary	Yes	Female	30	HR+/HER2+	Positive	Positive	Positive	Positive	Positive
P-0007497	P-0007497-T01-IM5	Metastasis	Skin	Yes	Female	55	HR-/HER2+	Negative	Negative	Negative	Positive	Positive
P-0007685	P-0007685-T01-IM5	Primary	Treatment Naive Primary	Yes	Female	50	HR+/HER2+	Positive	Positive	Positive	Positive	Positive
P-0007808	P-0007808-T01-IM5	Metastasis	Liver	Yes	Female	83	HR-/HER2+	Negative	Negative	Negative	Positive	Negative
P-0008010	P-0008010-T01-IM5	Metastasis	Liver	Yes	Female	41	HR+/HER2+	Positive	Positive	Positive	Positive	Positive
P-0008171	P-0008171-T01-IM5	Primary	Treatment Naive Primary	Yes	Female	53	HR+/HER2+	Positive	Positive	Positive	Positive	Negative
P-0008469	P-0008469-T01-IM5	Metastasis	Lung	Yes	Female	40	HR+/HER2+	Positive	Positive	Positive	Positive	Negative
P-0008482	P-0008482-T01-IM5	Primary	Treatment Naive Primary	Yes	Female	68	HR-/HER2+	Negative	Negative	Negative	Positive	Positive
P-0008559	P-0008559-T01-IM5	Primary	Treatment Naive Primary	Yes	Female	37	HR+/HER2+	Positive	Positive	Positive	Positive	Positive
P-0008572	P-0008572-T01-IM5	Metastasis	Skin	Yes	Female	73	HR+/HER2+	Positive	Positive	Positive	Positive	Positive
P-0008653	P-0008653-T01-IM5	Metastasis	Lymph Node	Yes	Female	56	HR+/HER2+	Positive	Positive	Positive	Positive	Positive
P-0008839	P-0008839-T01-IM5	Metastasis	Chest Wall	Yes	Female	56	HR-/HER2+	Negative	Negative	Negative	Positive	Positive
P-0008856	P-0008856-T01-IM5	Primary	Post-Treatment Primary	Yes	Female	47	HR+/HER2+	Positive	Positive	Positive	Positive	Positive
P-0008931	P-0008931-T01-IM5	Metastasis	Liver	Yes	Female	39	HR+/HER2+	Positive	Positive	Positive	Positive	Negative
P-0009014	P-0009014-T01-IM5	Primary	Post-Treatment Primary	Yes	Female	45	HR-/HER2+	Negative	Negative	Negative	Positive	Positive
P-0010095	P-0010095-T01-IM5	Metastasis	cal Recurrence/Lymph Nc	Yes	Female	57	HR+/HER2+	Positive	Positive	Negative	Positive	Negative
P-0010809	P-0010809-T01-IM5	Metastasis	Chest Wall	Yes	Female	50	HR-/HER2+	Negative	Negative	Negative	Positive	Positive
P-0012889	P-0012889-T01-IM5	Metastasis	Lymph Node	Yes	Female	48	HR+/HER2+	Positive	Positive	Positive	Positive	Negative
P-0013154	P-0013154-T01-IM5	Metastasis	Liver	Yes	Female	35	HR+/HER2+	Positive	Positive	Negative	Positive	Negative
P-0013385	P-0013385-T01-IM5	Metastasis	Liver	Yes	Female	49	HR+/HER2+	Positive	Positive	Positive	Positive	Negative
P-0014244	P-0014244-T01-IM6	Metastasis	Liver	Yes	Female	36	HR+/HER2+	Positive	Positive	Positive	Positive	Negative
P-0014435	P-0014435-T01-IM6	Metastasis	Liver	Yes	Female	25	HR+/HER2+	Positive	Positive	Positive	Positive	Positive
P-0014842	P-0014842-T01-IM6	Metastasis	Chest Wall	Yes	Female	64	HR+/HER2+	Positive	Positive	Positive	Positive	Negative
P-0015178	P-0015178-T01-IM6	Metastasis	Brain	Yes	Female	49	HR+/HER2+	Positive	Positive	Positive	Positive	Negative
P-0015408	P-0015408-T01-IM6	Metastasis	Lung	Yes	Female	46	HR+/HER2+	Positive	Negative	Positive	Positive	Unk/ND
P-0016179	P-0016179-T01-IM6	Metastasis	Lung	Yes	Female	49	HR+/HER2+	Positive	Positive	Positive	Positive	Equivocal

Positive	THP->HP	22034	22944	22944	910	29.9	1	1	0	0	0	1
Positive	Carboplatin-Paclitaxel-Trastuzumab	21685	24364		2679	88.1	0	0	0	0	0	0
Positive	THP->HP	19409	19892		483	15.9	0	1	0	0	0	1
Positive	THP->HP	19739	21878		2139	70.3	0	1	0	0	0	1
Positive	Carboplatin-Docetaxel-Trastuzumab	13635	14512		877	28.8	0	1	0	0	0	1
Unk/ND	THP	15748	17436		1688	55.5	0	0	0	0	0	0
Negative	THP->Exemestane-HP	15223	16039		816	26.8	0	1	0	0	0	1
Positive	THP->HP	15135	16660		1525	50.1	0	0	0	0	0	0
Positive	Tamoxifen-HP	23088	23102		14	0.5	0	0	0	0	0	0
Positive	THP	22084	24033		1949	64.1	0	0	0	0	0	0
Positive	THP	21026	21278		252	8.3	0	1	0	0	0	1
Positive	AC-TH	12428	13877	13877	1449	47.6	1	0	0	0	0	0
Positive	THP->HP	25894	26483		589	19.4	0	1	0	0	0	1
Positive	Vinorelbine-Trastuzumab	16251	16593		342	11.2	0	1	0	0	0	1
Positive	Letrozole-Trastuzumab	20163	21464		1301	42.8	0	0	0	0	0	0
Positive	THP->HP	12742	14007		1265	41.6	0	0	0	0	0	0
Positive	TH->Trastuzumab	12788	17059	17059	4271	140.4	1	0	0	0	0	0
Positive	THP	24789	25524	25524	735	24.2	1	1	0	0	0	1
Positive	THP->HP	19617	21282		1665	54.7	0	0	0	0	0	0
Negative	THP->Tamoxifen-HP	17007	18624		1617	53.2	0	0	0	0	0	0
Positive	THP	17445	18167		722	23.7	0	0	0	0	0	0
Positive	Capecitabine-Trastuzumab	21867	24269	24269	2402	79.0	1	0	1	0	0	1
Unk/ND	Vinorelbine-Trastuzumab	14102	14750		648	21.3	0	0	0	0	1	1
Positive	TH->Trastuzumab	18254	20822		2568	84.4	0	1	0	0	0	1
Positive	TDM1	16544	16775		231	7.6	0	1	0	0	0	1
Positive	THP->HP	15883	17637		1754	57.7	0	1	0	0	0	1
Negative	Exemestane-HP	21013	21444	21444	431	14.2	1	0	0	0	0	0
Positive	THP->HP	13821	15104		1283	42.2	0	0	0	0	0	0
Positive	THP	20944	21345	21345	401	13.2	1	1	0	0	0	1
Positive	THP	23759	24084		325	10.7	0	0	0	0	0	0
Positive	THP	12065	13108		1043	34.3	0	0	1	0	0	1
Positive	THP->HP	19437	20852		1415	46.5	0	0	0	0	0	0
Positive	THP->HP	24863	25479		616	20.3	0	1	0	0	0	1
Positive	THP	16121	18122		2001	65.8	0	0	0	0	0	0
Positive	Gemcitabine-Trastuzumab	24048	24310		262	8.6	0	1	0	0	0	1
Negative	THP	18443	19853		1410	46.4	0	1	0	0	0	1
Positive	THP	20128	20688		560	18.4	0	0	0	0	0	0
Positive	THP	20795	21877		1082	35.6	0	0	0	0	0	0
Positive	THP	27509	28678	28678	1169	38.4	1	1	0	0	0	1
Positive	Tamoxifen-Trastuzumab	11379	11999		620	20.4	0	0	0	0	0	0
Positive	THP	27232	27435		203	6.7	0	1	0	0	0	1
Negative	TH	9016	10204	10204	1188	39.1	1	1	0	0	0	1
Positive	THP->HP	21853	22953		1100	36.2	0	0	1	0	0	1
Positive	THP	11025	11819		794	26.1	0	0	0	0	0	0
Positive	TH	16795	20044		3249	106.8	0	0	0	0	0	0
Positive	Fulvestrant-Trastuzumab	15838	18432	18432	2594	85.3	1	1	0	0	0	1
Positive	THP	21911	23063	23063	1152	37.9	1	0	0	0	0	0
Unk/ND	THP	21772	22185	22185	413	13.6	1	1	1	0	0	1
Unk/ND	THP	25362	27037	27037	1675	55.1	1	0	0	0	0	0
Positive	TH	13387	16342		2955	97.2	0	0	0	0	0	0
Positive	THP->HP	10281	10841		560	18.4	0	0	0	0	0	0
Positive	THP->HP	25153	25681		528	17.4	0	0	0	0	1	1
Negative	Vinorelbine-HP	16435	16747	16747	312	10.3	1	0	0	0	0	0
Positive	THP->Tamoxifen-HP	11051	11799		748	24.6	0	0	0	0	0	0
Positive	TH	20118	23470	23470	3352	110.2	1	1	0	0	0	1
Positive	THP->Anastrozole-HP	18424	19097		673	22.1	0	0	0	0	0	0
Positive	THP->HP	31650	31973	31973	323	10.6	1	1	0	0	0	1
Positive	THP->HP	15078	15590		512	16.8	0	0	0	0	0	0
Negative	THP	20828	21264	21264	436	14.3	1	1	0	0	0	1
Positive	Fulvestrant-Trastuzumab	16907	17378		471	15.5	0	1	0	0	0	1
Positive	THP->HP	24919	25436		517	17.0	0	0	0	0	0	0
Positive	THP->Letrozole-HP	13539	13721		182	6.0	0	1	0	0	0	1
Positive	Gemcitabine-HP	27790	28259		469	15.4	0	1	0	0	0	1
Positive	THP->HP	20495	20983		488	16.0	0	0	0	0	0	0
Positive	THP->HP	20915	21299		384	12.6	0	0	0	0	0	0
Positive	THP	17160	18107		947	31.1	0	1	0	0	0	1
Negative	Capecitabine-Trastuzumab	14953	15316		363	11.9	0	1	0	0	0	1
Positive	THP->HP	16359	17531		1172	38.5	0	0	0	0	0	0
Negative	Gemcitabine-Lapatinib	22627	22764		137	4.5	0	1	0	0	0	1
Positive	THP->HP	18717	19706		989	32.5	0	1	0	0	0	1
Positive	THP	18876	19497		621	20.4	0	1	0	0	0	1
Negative	Carboplatin-HP	14652	15305		653	21.5	0	0	0	0	0	0
Positive	THP->Anastrozole-HP	19762	19977		215	7.1	0	0	0	0	1	1
Positive	Letrozole-Trastuzumab	13096	13258		162	5.3	0	0	0	0	0	0
Negative	Tamoxifen-Trastuzumab	9220	11481		2261	74.3	0	0	0	0	0	0
Positive	Capecitabine-HP	24215	24232		17	0.6	0	1	0	0	0	1
Positive	Capecitabine-HP	19357	19879		522	17.2	0	0	0	0	0	0
Positive	Anastrozole-Trastuzumab	23661	25012		1351	44.4	0	1	0	0	0	1
Positive	Letrozole-Trastuzumab	21764	23848		2084	68.5	0	0	0	0	0	0

Table S1 Patient-level clinical data and treatment data for this study cohort extracted from Razavi et al. 2018 Cancer Cell

<table>
<tr><th>Variable Name</th><th>Field Label</th><th>Values</th><th>Reference</th></tr>
<tr><td>Patient_ID</td><td>ID assigned to the patient.</td><td></td><td>Razavi et al. Table S2</td></tr>
<tr><td>Sample_ID</td><td>ID assigned to the sample.</td><td></td><td>Razavi et al. Table S2</td></tr>
<tr><td>Sample_Type</td><td>Site of tumor sequencing.</td><td>Metastasis | Primary</td><td>Razavi et al. Table S2</td></tr>
<tr><td>Sample_Site</td><td>Detailed description of the tissue site used for tumor sequencing.</td><td>Primary Treatment Naive | Primary Post-NACT: Primary tumor sample collected after exposure to neoadjuvant therapy | Primary Post-treatment: De novo metastatic primary or a second primary that have been exposed to treatment prior to sample collection for sequencing| Axillary Lymph Nodes Post NACT (Neoadjuvant Chemotherapy) | Local Recurrence Breast | Local Recurrence Axilla (LN) | Local Recurrence Chest Wall | Contralateral Breast | Liver | Bone | Chest Wall | Brain | Dura (Meninges) | Epidural Mass | Orbit | Parotid | Lymph Node | Lung | Pericardium | Pleura | Trachea | Pleural Fluid | Stomach | Bowel | Esophegus | Peritoneum | Ascites Fluid | Retroperitoneum | Ovary | Uterus | Cervix | Bladder | Ureter | Skin | Soft Tissue | Muscle</td><td>Razavi et al. Table S2</td></tr>
<tr><td>Metastatic_Dz</td><td>Indicate metastatic disease at last followup.</td><td>Yes | No</td><td>Razavi et al. Table S2</td></tr>
<tr><td>Gender</td><td>Biologic sex of patient.</td><td>Male | Female</td><td>Razavi et al. Table S2</td></tr>
<tr><td>Invasive_Carcinoma_Dx_Age</td><td>Age at diagnosis in years.</td><td></td><td>Razavi et al. Table S2</td></tr>
<tr><td>Receptor_Status_Patient</td><td>Overall patient breast cancer receptor subtype based on the reivew of the primary and metastatic tumors and the clinical history.</td><td>HR+/HER2- | HR+/HER2+ | HR-/HER2+ | Triple Negative</td><td>Razavi et al. Table S2</td></tr>
<tr><td>ER_Status_Patient</td><td>Overall patient breast cancer estrogen receptor status based on the reivew of the primary and metastatic tumors.</td><td>Positive | Negative</td><td>Based on ER_Status_Parimary and ER_Status_Sample</td></tr>
<tr><td>ER_Status_Primary</td><td>ER status of the primary tumor per pathology report.</td><td>Positive | Negative | Unk/ND: Unknown/Not Done</td><td>Razavi et al. Table S2</td></tr>
<tr><td>ER_Status_Sample</td><td>ER status of the sample underwent sequencing per pathology report.</td><td>Positive | Negative | Unk/ND: Unknown/Not Done</td><td>Razavi et al. Table S2</td></tr>
<tr><td>HER2_Status_Patient</td><td>Overall patient breast cancer HER2 status based on the reivew of the primary and metastatic tumors and the clinical history.</td><td>Positive | Negative</td><td>Razavi et al. Table S2</td></tr>
<tr><td>HER2_Status_Primary</td><td>Oerall HER2 status of the primary tumor.</td><td>Positive | Negative | Equivocal | Unk/ND: Unknown/Not Done</td><td>Razavi et al. Table S2</td></tr>
<tr><td>HER2_Status_Sample</td><td>Overall HER2 status of the sample underwent sequencing per pathology report.</td><td>Positive | Negative | Equivocal | Unk/ND: Unknown/Not Done</td><td>Razavi et al. Table S2</td></tr>
<tr><td>Treatment_Regimen</td><td>Treatment regimen received</td><td>Abbreviations: AC-TH: Doxorubicin-Cyclophosphamide, followed by a Taxane-Trastuzumab; HP: Trastuzumab-Pertuzumab; TH: Taxane-Trastuzumab; THP: Taxnane-Trastuzumab-Pertuzumab;</td><td>Razavi et al. Table S2</td></tr>
<tr><td>Anti-HER2-Treatment Start_Time</td><td>Time the regimen began in days (reference: date of birth)</td><td></td><td>Razavi et al. Table S2</td></tr>
<tr><td>Last_Contact_Time</td><td>Time of the last follow up in days (reference: date of birth).</td><td></td><td>Razavi et al. Table S2</td></tr>
<tr><td>Death_Time</td><td>Time of death in days (reference: date of birth).</td><td></td><td>Razavi et al. Table S2</td></tr>
<tr><td>OS_Day</td><td>Overall survival from the time of initial diagnosis in days.</td><td></td><td>Razavi et al. Table S2</td></tr>
<tr><td>OS_Month</td><td>Overall survival from the time of initial diagnosis in months.</td><td></td><td>Razavi et al. Table S2</td></tr>
<tr><td>OS_Event</td><td>Overall survival event.</td><td>1= Dead</td><td>Razavi et al. Table S2</td></tr>
<tr><td>PIK3CA_mutation</td><td>Mutation status of PIK3CA</td><td>1= Mutated</td><td>Razavi et al. Table S3</td></tr>
<tr><td>PIK3R1_mutation</td><td>Mutation status of PIK3R1</td><td>1= Mutated</td><td>Razavi et al. Table S3</td></tr>
<tr><td>AKT1_mutation</td><td>Mutation status of AKT1</td><td>1= Mutated</td><td>Razavi et al. Table S3</td></tr>
<tr><td>PTEN_loss_or_mutation</td><td>Mutation status of PTEN</td><td>1= Loss or Mutated</td><td>Razavi et al. Table S3</td></tr>
<tr><td>PI3K_Pathway_Mutation_Status</td><td>Mutation status of PI3K_Pathway based on the mutation statsu of PIK3CA, PIK3R1, AKT1 and PTEN.</td><td>1= Mutated</td><td>Based on PIK3CA_Mutation, PIK3R1_Mutation, AKT1_Mutation, and PTEN_Loss_or_Mutation</td></tr>
</table>

Supplementary Table S1. The clinical dataset of HER2+ metastatic or recurrent breast cancer patients treated with anti-HER2 therapies extracted from Razavi et al. 2018 Cancer Cell (related to main figure 1).

The clinical data and the information of PI3K pathway status of extracted patients are displayed.
